# Supplementary material for: Integrated omics endotyping of infants with respiratory syncytial virus bronchiolitis and risk of childhood asthma
Source: Nat Commun. 2021 Jun 14;12:3601. doi: 10.1038/s41467-021-23859-6 (PMC8203688; doi:10.1038/s41467-021-23859-6)

SUPPLEMENTARY INFORMATION

Integrated omics endotyping of infants with respiratory syncytial virus bronchiolitis and risk of childhood asthma

Authors: Yoshihiko Raita, Marcos Pérez-Losada, Robert J. Freishtat, Brennan Harmon, Jonathan M. Mansbach, Pedro A. Piedra, Zhaozhong Zhu, Carlos A. Camargo, Jr., and Kohei Hasegawa

CONTENTS

Supplementary Table 1. Comparisons between analytic and non-analytic cohorts in infants with respiratory syncytial virus infection in the MARC-35 cohort……………..…………………..……..……3

Supplementary Table 2. Between-endotype differences in nasopharyngeal metabolome data in infants with respiratory syncytial virus bronchiolitis……………...…………………………………..……..……5

Supplementary Table 3. Clinical, virus, nasopharyngeal microbiome, and metabolome data in the endotype A vs. B comparison…………………………………………………………….....……….7

Supplementary Table 4. Differentially-expressed genes in the endotype A vs. B comparison……......10

Supplementary Table 5. Association of respiratory syncytial virus bronchiolitis endotypes in infants excluding rhinovirus coinfection with development of asthma and recurrent wheeze outcomes………12

Supplementary Table 6. Association of five respiratory syncytial virus bronchiolitis endotypes in infants with development of asthma and recurrent wheeze outcomes using all variables……….........13

Supplementary Table 7. Sensitivity analysis using five endotypes…………….…….……………..…14

Supplementary Table 8. Association of five respiratory syncytial virus bronchiolitis endotypes in infants with development of asthma and recurrent wheeze outcomes using five endotypes……………17

Supplementary Table 9. Primers for respiratory pathogen testing………………………………………..18

Supplementary Table 10. Chromatography condition for metabolome profiling………………….. . .20

Supplementary Table 11. Principal investigators at the 17 participating sites in MARC-35……........22

Supplementary Fig. 1. Study flow diagram…………………….………….……………...………23

Supplementary Fig. 2. Average silhouette score and network modularity, according to number of endotypes………...………………………………………………………………………....…24

Supplementary Fig. 3. Similarity network visualization of respiratory syncytial virus bronchiolitis endotypes………………………………………………………………………….………..…25

Supplementary Fig. 4. Differential gene expression analysis and functional pathway analysis in the endotype A vs. C comparison……………………...……………………………………………….27

Supplementary Fig. 5. Differential gene expression analysis and functional pathway analysis in the endotype A vs. D comparison……………………...……………………………………...…..29

Supplementary Fig. 6. Ranking of normalized mutual information score…………………...………31

Supplementary Fig. 7. Alluvial plot that examines consistencies between original analysis and analysis using all variables……………………………..…………………………………………………32

Supplementary Fig. 8. Alluvial plot that examines consistencies across different number of endotypes………………………………………………………………………………………...33

Supplementary Fig. 9. Differential gene expression analysis and functional pathway analysis in the endotype 1 vs. 2 comparison in sensitivity analysis (corresponding to endotype A vs B in the primary analysis)……………………………………………………………………………………….34

Supplementary Table 1. Comparisons between analytic and non-analytic cohorts in infants with respiratory syncytial virus infection in the MARC-35 cohort

Characteristics	Analytic cohort (n=221)	Non-analytic cohort (n=531)	P-value*
**Demographics**			
Age (month), median (IQR)	3 (2–6)	3 (2–5)	0.65
Female sex	92 (41.6)	215 (40.5)	0.84
Race/ethnicity			0.52
Non-Hispanic white	92 (41.6)	252 (47.5)	
Non-Hispanic black	54 (24.4)	113 (21.3)	
Hispanic	66 (29.9)	144 (27.1)	
Other or unknown	9 (4.1)	22 (4.1)	
Prematurity (32–37 weeks)	46 (20.8)	97 (18.3)	0.48
Birth weight (kg), median (IQR)	3.20 (2.85–3.54)	3.30 (2.90–3.60)	0.045
Mode of birth (caesarean delivery)	75 (34.6)	179 (34.0)	0.96
Previous breathing problems (count)			0.59
0	189 (85.5)	439 (82.7)	
1	24 (10.9)	72 (13.6)	
2	8 (3.6)	20 (3.8)	
Previous ICU admission	4 (1.8)	7 (1.3)	0.74
Lifetime antibiotic use	67 (30.3)	165 (31.1)	0.91
Ever attended day care	66 (29.9)	112 (21.1)	0.01
Cigarette smoke exposure at home	32 (14.5)	80 (15.1)	0.93
Maternal smoking during pregnancy	30 (13.8)	72 (13.7)	0.99
Parental history of asthma	68 (30.8)	174 (32.8)	0.64
Parental history of eczema	41 (18.6)	97 (18.3)	0.99
**Clinical presentation**			
Weight at presentation (kg), median (IQR)	5.90 (4.60–7.90)	5.90 (4.80–7.41)	0.96
Respiratory rate at presentation (per minute), median (IQR)	48 (40–60)	50 (40–60)	0.53
Oxygen saturation at presentation			0.17
<90%	27 (12.6)	84 (16.2)	
90–93%	173 (80.5)	384 (74.0)	
≥94%	15 (7.0)	51 (9.8)	
Blood eosinophilia (≥4%)	18 (9.7)	40 (8.6)	0.77
IgE sensitization (%)	46 (20.8)	102 (19.2)	0.69
**Respiratory virus**			
RSV solo infection	158 (71.5)	383 (72.1)	0.93
Rhinovirus coinfection	29 (13.1)	80 (15.1)	0.57
Rhinovirus-A	14 (6.3)	37 (7.0)	0.88
Rhinovirus-B	4 (1.8)	8 (1.5)	0.99
Rhinovirus-C	11 (5.0)	33 (6.2)	0.63

Abbreviations: IQR, interquartile range; ICU, intensive care unit; IgE, immunoglobulin E

Data are no. (%) of infants unless otherwise indicated. Percentages may not equal 100, because of rounding and missingness.
* Two-sided raw P-values.

Supplementary Table 2. Between-endotype differences in nasopharyngeal metabolome data in infants with respiratory syncytial virus bronchiolitis

Metabolite intensity*	Endotype A (n=43; 19.5%)	Endotype B (n=63; 28.5%)	Endotype C (n=63; 28.5%)	Endotype D (n=52; 23.5%)	P-value†	FDR
Dihomolinolenate (20:3n3 or 3n6)	0.51 (-0.31– 1.14)	-0.36 (-2.15– 1.39)	0.65 (-0.53– 1.65)	-0.43 (-0.98– 0.44)	0.02	0.12
Uracil	-0.07 (-1.08– 1.59)	0.39 (-0.86– 1.39)	0.90 (-0.20– 1.84)	-0.68 (-1.28– 0.03)	<0.001	0.004
Xanthine	0.77 (0.03– 1.81)	0.81 (-0.24– 1.61)	1.06 (0.52– 1.68)	0.29 (-0.37– 0.87)	<0.001	0.018
Mannitol/sorbitol	-0.07 (-0.38– 0.72)	0.18 (-0.46– 1.94)	0.58 (-0.11– 1.93)	-0.01 (-0.72– 0.43)	0.003	0.052
Uridine	0.57 (-0.01– 1.10)	0.31 (-0.40– 1.17)	0.74 (0.28– 1.19)	0.04 (-0.40– 0.65)	0.001	0.024
3-hydroxybutyrate (BHBA)	0.01 (-1.12– 1.42)	0.13 (-0.95– 2.04)	1.15 (0.04– 2.97)	-0.50 (-1.83– 0.56)	<0.001	0.004
Glycylvaline	0.32 (-2.03– 1.13)	-0.36 (-2.72– 0.57)	-0.14 (-2.72– 0.76)	-1.23 (-3.21– -0.06)	0.03	0.14
Gulonate	0.66 (-0.27– 1.02)	0.57 (-0.44– 1.17)	0.95 (0.31– 1.49)	0.04 (-0.68– 0.60)	<0.001	0.004
1-(1-enyl-stearoyl)-GPE (P-18:0)	0.54 (-0.70– 1.47)	-0.24 (-1.43– 1.39)	0.85 (-0.15– 1.76)	-0.53 (-1.59– 0.55)	0.001	0.024
Urate	-0.17 (-0.84– 0.97)	-0.08 (-1.49– 0.77)	0.87 (-0.14– 1.57)	-0.10 (-1.12– 0.68)	0.001	0.024
Hypoxanthine	0.90 (0.31– 1.39)	0.32 (-0.29– 1.53)	0.94 (0.50– 1.49)	0.43 (-0.09– 1.00)	0.009	0.091
1-stearoyl-GPG (18:0)	-0.13 (-1.40– 1.01)	-1.24 (-3.62– 0.51)	-0.04 (-2.11– 1.40)	-2.24 (-3.62– 0.20)	0.008	0.086
3-indoxyl sulfate	-1.55 (-3.57– 0.58)	-3.57 (-3.57– -0.21)	-0.90 (-3.57– 0.06)	-2.60 (-3.57– 0.62)	0.26	0.45
Sphingomyelin (d18:1/14:0, d16:1/16:0)	0.81 (-0.01– 1.41)	0.55 (-0.18– 1.79)	0.82 (0.11– 1.68)	0.67 (-0.52– 1.36)	0.44	0.61
Sphingomyelin (d18:1/20:0, d16:1/22:0)	0.57 (-0.04– 1.30)	-0.04 (-1.18– 1.41)	0.64 (-0.15– 1.31)	0.32 (-0.86– 1.32)	0.22	0.41
Adenine	0.11 (-0.83– 1.60)	0.49 (-1.28– 1.78)	0.42 (-0.49– 1.88)	-0.57 (-1.48– 0.46)	0.005	0.074
1-stearoyl-GPI (18:0)	0.67 (-0.01– 1.44)	0.23 (-0.94– 1.39)	1.00 (-0.18– 1.91)	-0.09 (-1.19– 0.65)	0.001	0.024
N-acetylglucosamine/N-acetylgalactosamine	-0.88 (-4.20– 0.48)	-2.13 (-4.20– 0.17)	-0.75 (-3.78– 0.77)	-3.62 (-4.20– -0.73)	0.01	0.11
Sedoheptulose	0.43 (-0.26– 1.13)	0.25 (-1.09– 1.08)	0.64 (0.00– 1.34)	-0.24 (-1.13– 0.46)	0.005	0.074
Glycerophosphoethanolamine	0.21 (-0.18– 1.09)	0.10 (-0.71– 1.14)	0.56 (0.02– 1.10)	-0.01 (-0.68– 0.80)	0.04	0.15
Lactosyl-N-palmitoyl-sphingosine (d18:1/16:0)	0.56 (-0.03– 1.98)	1.04 (0.02– 2.01)	1.16 (0.27– 2.08)	0.42 (-0.41– 1.19)	0.03	0.14
1-stearoyl-2-linoleoyl-GPS (18:0/18:2)	0.56 (-0.21– 1.58)	0.85 (-0.08– 1.70)	0.71 (0.22– 1.97)	0.05 (-0.66– 1.16)	0.01	0.097
Sphingomyelin (d18:1/24:1, d18:2/24:0)	0.64 (0.19– 1.34)	0.46 (-0.34– 1.89)	0.85 (0.17– 1.52)	0.46 (-0.72– 1.21)	0.26	0.46
1-stearoyl-2-linoleoyl-GPC (18:0/18:2)	0.52 (0.06– 1.10)	0.46 (-0.75– 1.77)	0.65 (-0.82– 1.37)	0.58 (-0.63– 1.44)	0.99	0.99
1-stearoyl-GPE (18:0)	0.61 (-0.25– 1.47)	0.40 (-1.14– 1.52)	1.00 (0.15– 1.87)	0.35 (-0.66– 1.02)	0.03	0.13
Nicotinate	-1.90 (-4.68– 0.75)	-0.87 (-4.68– 0.93)	-4.68 (-4.68– -1.19)	-4.58 (-4.68– -0.56)	<0.001	0.022
Inosine	0.11 (-0.78– 0.81)	0.18 (-2.81– 0.88)	0.11 (-0.99– 1.39)	-0.71 (-3.78– 0.39)	0.022	0.13
1-(1-enyl-stearoyl)-2-linoleoyl-GPE (P-18:0/18:2)	0.58 (0.06– 2.03)	1.03 (-0.27– 2.07)	1.15 (0.23– 2.06)	0.06 (-0.57– 1.30)	0.02	0.13
Sphinganine	0.65 (0.11– 1.96)	1.06 (-0.10– 2.19)	0.96 (0.41– 2.33)	0.08 (-0.83– 1.22)	0.009	0.09

Prolylhydroxyproline	0.63 (-0.63– 1.38)	-0.35 (-2.69– 0.85)	0.29 (-1.27– 1.01)	0.52 (-0.64– 1.06)	0.05	0.18

Abbreviations: FER, false discovery rate; IQR, interquartile range; BHBA, beta-Hydroxybutyric acid; GPE, glycerophosphoethanolamine; GPG, glycerophosphoglycerol; GPI, Glycosylphosphatidylinositol; GPS, glycerophosphatidylserine; GPC, glycerophosphorylcholine

[*] For the metabolome data, the median (IQR) intensity of 30 metabolites with the highest normalized mutual information score, after processing by log_2 transformation with batch effect adjustment.
[†] Two-sided raw P-values.

Supplementary Table 3. Clinical, virus, nasopharyngeal microbiome, and metabolome data in the endotype A vs. B comparison

Characteristics	Endotype A (n=43; 19.5%)	Endotype B (n=63; 28.5%)	P-value*	FDR
**Demographics**				
Age (month), median (IQR)	2 (1–4)	4 (2–6)	0.006	
Female sex	35 (81.4)	30 (47.6)	0.001	
Race/ethnicity			0.13	
Non-Hispanic white	21 (48.8)	17 (27.0)		
Non-Hispanic black	9 (20.9)	20 (31.7)		
Hispanic	12 (27.9)	22 (34.9)		
Other or unknown	1 (2.3)	4 (6.3)		
Prematurity (32-37 weeks)	7 (16.3)	14 (22.2)	0.61	
Birth weight (kg), median (IQR)	3 (3–4)	3 (3–4)	0.37	
Mode of birth (caesarean delivery)	13 (30.2)	19 (30.6)	0.99	
Previous breathing problems (count)			0.76	
0	38 (88.4)	58 (92.1)		
1	3 (7.0)	4 (6.3)		
2	2 (4.7)	1 (1.6)		
Previous ICU admission	0 (0)	0 (0)	0.99	
Lifetime antibiotic use	2 (4.7)	12 (19.0)	0.04	
Ever attended daycare	13 (30.2)	21 (33.3)	0.90	
Cigarette smoke exposure at home	11 (25.6)	11 (17.5)	0.44	
Maternal smoking during pregnancy	8 (18.6)	6 (9.7)	0.30	
Parental history of asthma	6 (14.0)	50 (79.4)	<0.001	
Parental history of eczema	9 (20.9)	14 (22.2)	0.99	
**Clinical presentation**				
Weight (kg), median (IQR)	4.90 (4.14–5.65)	6.20 (5.18–7.75)	0.003	
Respiratory rate (per minute), median (IQR)	52 (41–61)	48 (40–62)	0.69	
Oxygen saturation			0.69	
<90%	5 (11.9)	4 (6.3)		
90-93%	35 (83.3)	55 (87.3)		
≥94%	2 (4.8)	4 (6.3)		
Blood eosinophilia (≥4%)	5 (13.5)	5 (9.8)	0.74	
IgE sensitization	7 (16.3)	15 (23.8)	0.49	

**Clinical course**				
Positive pressure ventilation use†	2 (4.7)	3 (4.8)	0.99	
Intensive treatment use‡	6 (14.0)	10 (15.9)	0.99	
Length-of-day (day), median (IQR)	2 (1–3)	2 (1–4)	0.20	
Antibiotic use during hospitalization	9 (20.9)	21 (33.3)	0.19	
Corticosteroid use during hospitalization	1 (2.3)	6 (9.5)	0.24	
**Respiratory virus**				
RSV solo infection	32 (74.4)	44 (69.8)	0.77	
Rhinovirus coinfection	5 (11.6)	13 (20.6)	0.34	
Rhinovirus-A	1 (2.3)	7 (11.1)	0.19	
Rhinovirus -B	2 (4.7)	1 (1.6)	0.74	
Rhinovirus -C	2 (4.7)	5 (7.9)	0.79	
**Chronic comorbidities**				
Asthma at age 5 years	4 (9.3)	24 (38.1)	0.001	
Recurrent wheeze by age 3 years	10 (23.3)	22 (34.9)	0.29	
Time to event (day), median (IQR)	1,095 (981–1,096)	1095 (459–1,095)	0.04	
**Microbiome relative abundance**§, median (IQR)				
Streptococcus pneumoniae	0.05 (0.02–0.38)	0.26 (0.11–0.47)	0.02	0.094
Moraxella catarrhalis	0.01 (0.00–0.09)	0.05 (0.00–0.22)	0.11	0.180
Moraxella nonliquefaciens	0.03 (0.00–0.73)	0.00 (0.00–0.07)	0.02	0.094
Cutibacterium acnes	0.01 (0.00–0.06)	0.01 (0.00–0.08)	0.90	0.90
Haemophilus influenzae	0.00 (0.00–0.01)	0.00 (0.00–0.04)	0.42	0.53
**Metabolome intensity ‖ , median (IQR)**				
N-acetylglutamine	-0.59 (-4.02– 0.04)	-0.08 (-1.38– 0.59)	0.018	0.9
Glutamine	-0.34 (-4.28– -0.03)	-0.00 (-0.56– 0.13)	0.035	0.9
Prolylhydroxyproline	0.63 (-0.63– 1.38)	-0.35 (-2.69– 0.85)	0.012	0.9
Spermidine	1.03 (0.34– 1.56)	0.38 (-0.36– 1.18)	0.013	0.9
Hydroxyproline	0.81 (0.05– 1.16)	0.42 (-0.48– 0.89)	0.043	0.9
N-acetylisoleucine	-0.53 (-0.98– 0.15)	0.06 (-0.96– 0.53)	0.022	0.9
N-acetylphenylalanine	-0.35 (-0.76– 0.16)	0.20 (-0.72– 0.53)	0.049	0.9

Abbreviations: IQR, interquartile range; ICU, intensive care unit; IgE, immunoglobulin E

Data are no. (%) of infants unless otherwise indicated. Percentages may not equal 100, because of rounding and missingness.

* Two-sided raw P-values.

† Infants with bronchiolitis who underwent continuous positive airway ventilation and/or mechanical ventilation.

‡ Infants with bronchiolitis who were admitted to ICU and/or who underwent positive pressure ventilation.

[§] For microbiome data, the five most abundant species are presented.
[‖] The metabolome data are processed by $\log_2$ transformation with batch effect adjustment and metabolites with a two-sided raw P-value <0.05 are presented.

Supplementary Table 4. Differentially-expressed genes in the endotype A vs. B comparison

ENSEMBL gene ID	Gene name	Log_2 fold change	$-log_{10}$ P-value*	FDR
ENSG00000158352	shroom family member 4	-1.16	7.25	<0.001
ENSG00000218336	teneurin transmembrane protein 3	-3.11	6.53	0.002
ENSG00000177854	transmembrane protein 187	-1.00	6.00	0.006
ENSG00000110852	C-type lectin domain family 2 member B	1.11	5.59	0.011
ENSG00000136928	gamma-aminobutyric acid type B receptor subunit 2	-2.93	4.84	0.034
ENSG00000178372	calmodulin like 5	-1.12	4.83	0.034
ENSG00000198829	succinate receptor 1	1.43	4.80	0.034
ENSG00000183828	nudix hydrolase 14	-0.62	4.70	0.037
ENSG00000006611	USH1 protein network component harmonin	-1.30	4.47	0.038
ENSG00000038945	macrophage scavenger receptor 1	0.83	4.41	0.038
ENSG00000134028	ADAM like decysin 1	0.77	4.42	0.038
ENSG00000169413	ribonuclease A family member k6	0.82	4.47	0.038
ENSG00000171195	mucin 7, secreted	1.63	4.44	0.038
ENSG00000173198	cysteinyl leukotriene receptor 1	0.95	4.47	0.038
ENSG00000175857	GRB2 binding adaptor protein, transmembrane	0.94	4.43	0.038
ENSG00000156234	C-X-C motif chemokine ligand 13	1.42	4.28	0.049
ENSG00000185862	ecotropic viral integration site 2B	1.07	4.26	0.049
ENSG00000159784	family with sequence similarity 131 member B	-1.06	4.20	0.052
ENSG00000064042	LIM and calponin homology domains 1	-0.70	4.01	0.077
ENSG00000265590	CFAP298-TCP10L readthrough	3.80	3.97	0.08
ENSG00000272921	Protein kinase domain-containing protein	4.26	3.93	0.09
ENSG00000196873	COBW domain containing 3	0.75	3.90	0.09

ENSG00000278705	H4 clustered histone 2	-0.74	3.86	0.09
ENSG00000154734	ADAM metallopeptidase with thrombospondin type 1 motif 1	-1.29	3.83	0.09
ENSG00000167654	ATCAY kinesin light chain interacting caytaxin	3.41	3.82	0.09
ENSG00000100003	SEC14 like lipid binding 2	-0.89	3.81	0.09
ENSG00000160201	U2 small nuclear RNA auxiliary factor 1	1.10	3.77	0.09
ENSG00000082781	integrin subunit beta 5	-0.69	3.77	0.09
ENSG00000124466	LY6/PLAUR domain containing 3	-0.77	3.77	0.09

Abbreviation: FDR, false discovery rate
These 29 differentially-expressed genes (FDR < 0.1 with ≥ |1.5|-fold change) are also shown in Fig. 5a (volcano plot).
* Two-sided raw P-values are used to calculate $-\log_{10}$ P-value.

Supplementary Table 5. Association of respiratory syncytial virus bronchiolitis endotypes in infants excluding rhinovirus coinfection with development of asthma and recurrent wheeze outcomes

**Endotypes**	**Childhood asthma at age 5 years***		**Recurrent wheeze by age 3 years† *with* asthma‡**		**Recurrent wheeze by age 3 years† *without* asthma§**	
	Odds ratio (95% CI)	P-value ‖	Hazard ratio (95% CI)	P-value‖	Hazard ratio (95% CI)	P-value‖
Endotype A (clinical^{classic}microbiome$^{M.\ nonliquefaciens}$inflammation$^{IFN\text{-}intermediate}$)	1 [Reference]	–	1 [Reference]	–	1 [Reference]	–
Endotype B (clinical^{atopic}microbiome$^{S.\ pneumoniae/M.\ catarrhalis}$inflammation$^{IFN\text{-}high}$)	5.21 (1.73–19.5)	0.006	4.61 (0.99–21.3)	0.051	1.14 (0.43–3.02)	0.80
Endotype C (clinical^{severe}microbiome^{Mixed}inflammation$^{IFN\text{-}low}$)	2.22 (0.70–8.47)	0.20	1.91 (0.37–9.82)	0.44	1.13 (0.46–2.77)	0.79
Endotype D (clinical$^{non\text{-}atopic}$microbiome$^{M.\ catarrhalis}$inflammation$^{IL\text{-}6}$)	2.36 (0.72–9.26)	0.18	3.35 (0.70–16.2)	0.13	1.20 (0.47–3.05)	0.70

Abbreviation: CI, confidential interval

[*] Asthma (binary outcome) was defined as physician-diagnosis of asthma by age 5 years, plus either asthma medication use (e.g., albuterol inhaler, inhaled corticosteroids, montelukast) or asthma-related symptoms in the preceding year. To examine the association between RSV bronchiolitis endotypes (endotype A as the reference) and the risk of developing childhood asthma, logistic regression model was fit (n=192).

[†] Recurrent wheeze (time-to-event outcome) was defined as having at least two corticosteroid-requiring exacerbations in six months or at least four wheezing episodes in one year that last at least one day and affect sleep. To examine the association between RSV bronchiolitis endotypes (endotype A as the reference) and the rate of recurrent wheeze, Cox proportional hazards model was fit.

[‡] The outcome is recurrent wheeze by age 3 years *with* epidemiological definition of asthma (n=23) vs. no recurrent wheeze or asthma (n=109). The analysis excludes the other children (n=89).

[§] The outcome is recurrent wheeze by age 3 years *without* epidemiological definition of asthma (n =38) vs. no recurrent wheeze or asthma (n=109). The analysis excludes the other children (n=74).

[‖] Two-sided raw P-values.

Supplementary Table 6. Association of respiratory syncytial virus bronchiolitis endotypes using all variables in infants with development of asthma and recurrent wheeze outcomes

**Endotypes**	**Childhood asthma at age 5 years***		**Recurrent wheeze by age 3 years† *with* asthma ‡**		**Recurrent wheeze by age 3 years† *without* asthma§**	
	Odds ratio (95% CI)	P-value‖	Hazard ratio (95% CI)	P-value‖	Hazard ratio (95% CI)	P-value‖
**Endotype 1**	1 [Reference]	–	1 [Reference]	–	1 [Reference]	–
**Endotype 2**	3.35 (1.35–9.19)	0.012	2.09 (0.70–6.24)	0.19	2.24 (0.90–5.55)	0.082
**Endotype 3**	1.20 (0.40–3.73)	0.74	0.97 (0.26–3.63)	0.97	1.61 (0.61–4.23)	0.33
**Endotype 4**	1.46 (0.54–4.26)	0.46	1.53 (0.50–4.67)	0.46	1.54 (0.61–3.92)	0.36

Abbreviation: CI, confidential interval

[*] Asthma (binary outcome) was defined as physician-diagnosis of asthma by age 5 years, plus either asthma medication use (e.g., albuterol inhaler, inhaled corticosteroids, montelukast) or asthma-related symptoms in the preceding year. To examine the association between RSV bronchiolitis endotypes (endotype A as the reference) and the risk of developing childhood asthma, logistic regression model was fit.

[†] Recurrent wheeze (time-to-event outcome) was defined as having at least two corticosteroid-requiring exacerbations in six months or at least four wheezing episodes in one year that last at least one day and affect sleep. To examine the association between RSV bronchiolitis endotypes (endotype A as the reference) and the rate of recurrent wheeze, Cox proportional hazards model was fit.

[‡] The outcome is recurrent wheeze by age 3 years *with* epidemiological definition of asthma (n=26) vs. no recurrent wheeze (n=127). The analysis excludes the other children (n=68).

[§] The outcome is recurrent wheeze by age 3 years *without* epidemiological definition of asthma (n =43) vs. no recurrent wheeze (n=127). The analysis excludes the other children (n=51).

[‖] Two-sided raw P-values.

Supplementary Table 7. Sensitivity analysis using five endotypes

	Endotype 1 (n=40)	Endotype 2 (n=49)	Endotype 3 (n=52)	Endotype 4 (n=40)	Endotype 5 (n=40)	P-value*
**Original 4 endotypes (A-D)**						
Endotype A (n=43): clinical^{classic}microbiome$^{M.\ nonliquefaciens}$inflammation$^{IFN\text{-}intermediate}$	36 (90.0)	0 (0)	3 (5.8)	2 (5.0)	2 (5.0)	
Endotype B (n=63): clinical^{atopic}microbiome$^{S.\ pneumoniae/M.\ catarrhalis}$inflammation$^{IFN\text{-}high}$	4 (10)	49 (100)	2 (3.8)	6 (15.0)	2 (5.0)	
Endotype C (n=63): clinical^{severe}microbiome^{Mixed}inflammation$^{IFN\text{-}low}$	0 (0)	0 (0)	47 (90.4)	0 (0)	16 (40.0)	
Endotype D (n=52): clinical$^{non\text{-}atopic}$microbiome$^{M.\ catarrhalis}$inflammation$^{IL\text{-}6}$	0 (0)	0 (0)	0 (0)	32 (80.0)	20 (50.0)	
**Demographics**						
Age (month), median (IQR)	2 (1–4)	4 (2–6)	6 (3–9)	4 (2–5)	2 (1-3)	<0.001
Female sex	32 (80.0)	26 (53.1)	16 (30.8)	8 (20.0)	10 (25.0)	<0.001
Race/ethnicity						0.46
Non-Hispanic white	18 (45.0)	13 (26.5)	27 (51.9)	19 (47.5)	15 (37.5)	
Non-Hispanic black	9 (22.5)	16 (32.7)	7 (13.5)	11 (27.5)	11 (27.5)	
Hispanic	12 (30.0)	17 (34.7)	16 (30.8)	8 (20.0)	13 (32.5)	
Other or unknown	1 (2.5)	3 (6.1)	2 (3.8)	2 (5.0)	1 (2.5)	
Prematurity (32–37 weeks)	7 (17.5)	11 (22.4)	9 (17.3)	14 (35.0)	5 (12.5)	0.15
Birth weight (kg), median (IQR)	3 (3–3)	3 (3–3)	3 (3–4)	3 (2–4)	3 (3–4)	0.03
Mode of birth (caesarean delivery)	11 (27.5)	17 (35.4)	20 (38.5)	13 (34.2)	14 (35.9)	0.87
Previous breathing problems (count)						0.27
0	36 (90.0)	44 (89.8)	40 (76.9)	36 (90.0)	33 (82.5)	
1	2 (5.0)	4 (8.2)	8 (15.4)	3 (7.5)	7 (17.5)	
2	2 (5.0)	1 (2.0)	4 (7.7)	1 (2.5)	0 (0)	
Previous ICU admission	0 (0)	0 (0)	2 (3.8)	0 (0)	2 (5.0)	0.20
Lifetime antibiotic use	1 (2.5)	10 (20.4)	44 (84.6)	4 (10.0)	8 (20.0)	<0.001
Ever attended day care	12 (30.0)	17 (34.7)	17 (32.7)	14 (35.0)	6 (15.0)	
Cigarette smoke exposure at home	11 (27.5)	10 (20.4)	5 (9.6)	5 (12.5)	1 (2.5)	0.01
Maternal smoking during pregnancy	7 (17.5)	5 (10.4)	8 (15.4)	7 (18.4)	3 (7.7)	0.56
Parental history of asthma	10 (25.0)	38 (77.6)	9 (17.3)	7 (17.5)	4 (10.0)	<0.001
Parental history of eczema	9 (22.5)	9 (18.4)	11 (21.2)	7 (17.5)	5 (12.5)	0.80
**Clinical Presentation**						
Weight at presentation (kg), median (IQR)	4.97 (4.10–5.93)	6.35 (5.30–7.7)	7.7 (5.65–8.68)	6.20 (4.85–7.77)	4.65 (4.27–6.14)	<0.001
Respiratory rate at presentation (per minute), median (IQR)	49 (42–60)	48 (40–64)	42 (38–53)	52 (43–62)	50 (44–60)	0.06

Oxygen saturation at presentation						
<90%	4 (10.3)	2 (4.1)	13 (26.0)	5 (12.8)	3 (7.9)	
90-93%	35 (89.7)	43 (87.8)	35 (70.0)	31 (79.5)	29 (76.3)	
≥94%	0 (0.0)	4 (8.2)	2 (4.0)	3 (7.7)	6 (15.8)	
Blood eosinophilia (≥4%)	5 (13.9)	5 (13.2)	2 (4.9)	4 (11.8)	2 (5.6)	0.52
IgE sensitization	7 (17.5)	8 (16.3)	16 (30.8)	11 (27.5)	4 (10.0)	0.10
**Clinical course**						
Positive pressure ventilation use†	1 (2.5)	3 (6.1)	5 (9.6)	0 (0.0)	8 (20.0)	0.008
Intensive treatment use ‡	6 (15.0)	9 (18.4)	9 (17.3)	3 (7.5)	10 (25.0)	0.32
Length of day (day), median (IQR)	2 (1–2)	3 (1–5)	2 (1–3)	2 (1–3)	3 (2–4)	0.08
Antibiotic use during hospitalization	8 (20.0)	17 (34.7)	24 (46.2)	6 (15.0)	16 (40.0)	0.006
Corticosteroid use during hospitalization	1 (2.5)	6 (12.2)	9 (17.3)	3 (7.5)	5 (12.5)	0.19
**Respiratory virus**						
RSV solo infection	30 (73.2)	33 (67.3)	31 (59.6)	32 (80.0)	32 (82.1)	0.11
Rhinovirus coinfection	6 (15.0)	11 (22.4)	2 (3.8)	5 (12.5)	5 (12.5)	0.08
Rhinovirus-A	1 (2.5)	6 (12.2)	2 (3.8)	1 (2.5)	4 (10.0)	0.22
Rhinovirus-B	2 (5.0)	1 (2.0)	0 (0.0)	1 (2.5)	0 (0.0)	0.35
Rhinovirus-C	3 (7.5)	4 (8.2)	0 (0.0)	3 (7.5)	1 (2.5)	0.16
**Chronic comorbidities**						
Asthma at age 5 years	5 (12.5)	20 (40.8)	10 (19.2)	9 (22.5)	7 (17.5)	0.02
Recurrent wheeze by age 3 years	10 (25.0)	18 (36.7)	14 (26.9)	13 (32.5)	14 (35.0)	0.71
Time to event (day), median (IQR)	1095 (1001–1096)	1095 (429–1095)	1095 (691–1096)	1095 (534–1096)	1095 (549–1096)	0.37
**Microbiome relative abundance§**, median (IQR)						
Streptococcus pneumoniae	0.17 (0.02–0.39)	0.24 (0.12–0.44)	0.12 (0.05–0.28)	0.04 (0.01–0.12)	0.39 (0.14–0.73)	<0.001
Moraxella catarrhalis	0.02 (0.00–0.09)	0.05 (0.00–0.22)	0.01 (0.00–0.12)	0.67 (0.15–0.90)	0.00 (0.00–0.03)	<0.001
Moraxella nonliquefaciens	0.06 (0.00–0.74)	0.00 (0.00–0.06)	0.00 (0.00–0.02)	0.00 (0.00–0.21)	0.00 (0.00–0.00)	0.001
Cutibacterium acnes	0.01 (0.00–0.089)	0.01 (0.00–0.07)	0.08 (0.01–0.19)	0.01 (0.00–0.02)	0.09 (0.04–0.27)	<0.001
Haemophilus influenzae	0.00 (0.00–0.00)	0.00 (0.00–0.04)	0.08 (0.00–0.42)	0.00 (0.00–0.00)	0.00 (0.00–0.00)	<0.001
**Metabolome intensity** ‖, median (IQR)						
Dihomolinolenate (20:3n3 or 3n6)	0.40 (-0.61– 1.12)	-0.31 (-2.21– 1.51)	1.03 (-0.08– 1.82)	-0.53 (-1.28– 0.71)	-0.35 (-1.21– 0.62)	0.001
Uracil	-0.17 (-1.93– 1.04)	0.12 (-0.93– 1.49)	1.55 (0.11– 2.51)	-0.48 (-1.05– 0.18)	-0.29 (-1.28– 0.94)	<0.001
Xanthine	0.64 (-0.14– 1.33)	0.72 (-0.37– 1.61)	1.19 (0.80– 1.77)	0.40 (-0.32– 0.88)	0.59 (-0.05– 1.29)	<0.001
Mannitol/sorbitol	-0.12 (-0.57– 0.61)	0.21 (-0.35– 1.79)	0.91 (0.04– 1.98)	0.02 (-0.61– 0.36)	0.19 (-0.58– 0.89)	0.004
Uridine	0.48 (-0.05– 0.99)	0.23 (-0.59– 1.13)	0.93 (0.61– 1.34)	0.17 (-0.35– 0.85)	0.29 (-0.40– 0.64)	<0.001

3-hydroxybutyrate (BHBA)	-0.15 (-1.29– 1.14)	0.20 (-0.94– 2.02)	1.73 (0.63– 3.23)	0.05 (-1.15– 1.39)	-0.52 (-1.74– 0.37)	<0.001
Glycylvaline	-0.25 (-2.32– 1.22)	-0.45 (-2.72– 0.18)	0.11 (-1.58– 1.06)	-1.02 (-2.85– -0.06)	-1.18 (-2.72– 0.25)	0.091
Gulonate	0.55 (-0.38– 1.01)	0.63 (-0.61– 1.08)	1.05 (0.32– 1.53)	0.10 (-0.75– 0.57)	0.54 (-0.23– 1.13)	<0.001
1-(1-enyl-stearoyl)-GPE (P-18:0)	0.38 (-0.68– 1.46)	-0.28 (-1.52– 1.64)	1.36 (0.23– 2.06)	-0.53 (-1.38– 0.36)	-0.23 (-1.44– 0.39)	<0.001
Urate	-0.16 (-1.47– 0.92)	-0.08 (-1.50– 0.73)	0.89 (-0.53– 1.65)	0.27 (-0.50– 0.78)	-0.30 (-1.14– 0.88)	0.008
Hypoxanthine	0.74 (0.20– 1.37)	0.32 (-0.58– 1.51)	1.00 (0.69– 1.64)	0.32 (-0.05– 1.05)	0.59 (-0.14– 1.15)	0.003
1-stearoyl-GPG (18:0)	-0.22 (-1.99– 0.99)	-1.24 (-3.62– 0.68)	0.12 (-0.78– 1.71)	-1.88 (-3.62– -0.06)	-1.87 (-3.62– 0.17)	<0.001
3-indoxyl sulfate	-1.79 (-3.57– -0.04)	-3.57 (-3.57– -0.14)	-1.24 (-3.57– -0.13)	-1.76 (-3.57– 0.59)	-3.57 (-3.57– 0.77)	0.18
Sphingomyelin (d18:1/14:0, d16:1/16:0)	0.53 (-0.02– 1.19)	0.34 (-0.36– 1.74)	1.26 (0.53– 1.79)	0.40 (-0.52– 1.40)	0.66 (-0.43– 1.34)	0.045
Sphingomyelin (d18:1/20:0, d16:1/22:0)	0.40 (-0.12– 1.06)	-0.09 (-1.34– 1.44)	0.70 (0.00– 1.31)	0.38 (-1.00– 1.39)	0.46 (-0.69– 1.33)	0.22
Adenine	0.10 (-1.21– 1.02)	0.46 (-1.34– 1.79)	1.11 (-0.00– 3.19)	-0.55 (-1.43– 0.92)	-0.40 (-1.74– 0.33)	<0.001
1-stearoyl-GPI (18:0)	0.57 (-0.08– 1.59)	0.23 (-1.02– 1.65)	1.25 (0.74– 2.25)	-0.20 (-1.39– 0.51)	-0.11 (-0.75– 0.75)	<0.001
N-acetylglucosamine/N-acetylgalactosamine	-1.76 (-4.20– 0.04)	-2.07 (-4.20– 0.15)	-0.34 (-1.65– 0.85)	-4.19 (-4.20– -0.65)	-3.02 (-4.20– -0.22)	0.001
Sedoheptulose	0.10 (-0.55– 0.89)	0.24 (-1.16– 1.23)	0.82 (0.18– 1.40)	-0.05 (-0.88– 0.46)	0.10 (-0.78– 0.95)	0.002
Glycerophosphoethanolamine	0.22 (-0.32– 0.96)	-0.17 (-0.91– 1.19)	0.77 (0.18– 1.30)	0.10 (-0.37– 1.02)	0.05 (-0.59– 0.59)	0.002
Lactosyl-N-palmitoyl-sphingosine (d18:1/16:0)	0.57 (-0.02– 2.11)	0.94 (-0.09– 1.83)	1.59 (0.57– 2.18)	0.12 (-0.54– 1.00)	0.65 (-0.16– 1.62)	0.003
1-stearoyl-2-linoleoyl-GPS (18:0/18:2)	0.55 (-0.26– 1.58)	0.60 (-0.39– 1.94)	1.25 (0.35– 2.02)	0.18 (-0.72– 0.97)	0.34 (-0.49– 1.54)	0.002
Sphingomyelin (d18:1/24:1, d18:2/24:0)	0.59 (0.13– 1.38)	0.44 (-0.63– 1.90)	1.10 (0.68– 1.62)	0.41 (-0.61– 1.02)	0.54 (-0.65– 1.52)	0.015
1-stearoyl-2-linoleoyl-GPC (18:0/18:2)	0.49 (0.04– 1.02)	0.38 (-0.76– 1.75)	0.65 (-0.52– 1.38)	0.62 (-0.63– 1.29)	0.61 (-0.56– 1.77)	0.98
1-stearoyl-GPE (18:0)	0.55 (-0.37– 1.45)	0.28 (-1.18– 1.50)	1.34 (0.60– 2.05)	0.10 (-0.66– 1.16)	0.43 (-0.49– 0.91)	0.001
Nicotinate	-2.55 (-4.68– 0.32)	-0.86 (-4.67– 0.91)	-4.68 (-4.68– -1.18)	-4.26 (-4.68– -0.66)	-4.58 (-4.68– -0.56)	0.005
Inosine	0.05 (-0.91– 0.86)	0.19 (-2.95– 0.85)	0.27 (-0.56– 1.41)	-0.53 (-3.78– 0.65)	-0.52 (-1.93– 0.51)	0.061
1-(1-enyl-stearoyl)-2-linoleoyl-GPE (P-18:0/18:2)	0.78 (0.09– 2.35)	0.83 (-0.57– 2.07)	1.50 (0.57– 2.11)	0.05 (-0.43– 1.19)	0.60 (-0.40– 1.56)	0.007
Sphinganine	0.57 (0.04– 1.77)	1.06 (-0.09– 2.18)	1.19 (0.47– 2.50)	0.08 (-0.77– 1.23)	0.61 (-0.71– 1.77)	0.003
Prolylhydroxyproline	0.32 (-0.87– 1.22)	-0.66 (-2.82– 0.54)	0.22 (-1.30– 0.92)	0.49 (-0.58– 1.27)	0.62 (-0.69– 1.78)	0.014

Abbreviations: IQR, interquartile range; ICU, intensive care unit; IgE, immunoglobulin E; BHBA, beta-Hydroxybutyric acid; GPE, glycerophosphoethanolamine; GPG, glycerophosphoglycerol; GPI, Glycosylphosphatidylinositol; GPS, glycerophosphatidylserine; GPC, glycerophosphorylcholine

Data are no. (%) of infants unless otherwise indicated. Percentages may not equal 100, because of rounding and missingness.

[*] Two-sided raw P-values.

[†] Infants with bronchiolitis who underwent continuous positive airway ventilation and/or mechanical ventilation.

[‡] Infants with bronchiolitis who were admitted to ICU and/or who underwent positive pressure ventilation.

[§] For microbiome data, the five most abundant species are presented.

[ǁ] For the metabolome data, the median (IQR) intensity of 30 metabolites with the highest normalized mutual information score, after processing by log_2 transformation with batch effect adjustment.

Supplementary Table 8. Association of five respiratory syncytial virus bronchiolitis endotypes in infants with development of asthma and recurrent wheeze outcomes using five endotypes

Endotypes	Childhood asthma at age 5 years*		Recurrent wheeze by age 3 years† *with* asthma ‡		Recurrent wheeze by age 3 years† *without* asthma§	
	Odds ratio (95% CI)	P-value‖	Hazard ratio (95% CI)	P-value‖	Hazard ratio (95% CI)	P-value‖
**Endotype 1** Concordant with endotype A	1 [Reference]	–	1 [Reference]	–	1 [Reference]	–
**Endotype 2** Concordant with endotype B	4.83 (1.72–16.0)	0.005	3.57 (0.97–13.2)	0.057	1.71 (0.64–4.61)	0.28
**Endotype 3** Concordant with endotype C	1.67 (0.54–5.77)	0.39	1.40 (0.33–5.85)	0.65	1.11 (0.41–2.99)	0.83
**Endotype 4**	2.03 (0.63–7.22)	0.24	2.25 (0.56–8.99)	0.25	1.17 (0.41–3.34)	0.77
**Endotype 5**	1.48 (0.43–5.45)	0.53	1.24 (0.25–6.17)	0.79	1.77 (0.69–4.57)	0.24

Abbreviation: CI, confidential interval

* Asthma (binary outcome) was defined as physician-diagnosis of asthma by age 5 years, plus either asthma medication use (e.g., albuterol inhaler, inhaled corticosteroids, montelukast) or asthma-related symptoms in the preceding year. To examine the association between RSV bronchiolitis endotypes (endotype A as the reference) and the risk of developing childhood asthma, logistic regression model was fit.

† Recurrent wheeze (time-to-event outcome) was defined as having at least two corticosteroid-requiring exacerbations in six months or at least four wheezing episodes in one year that last at least one day and affect sleep. To examine the association between RSV bronchiolitis endotypes (endotype A as the reference) and the rate of recurrent wheeze, Cox proportional hazards model was fit.

‡ The outcome is recurrent wheeze by age 3 years *with* epidemiological definition of asthma (n=26) vs. no recurrent wheeze (n=127). The analysis excludes the other children (n=68).

§ The outcome is recurrent wheeze by age 3 years *without* epidemiological definition of asthma (n =43) vs. no recurrent wheeze (n=127). The analysis excludes the other children (n=51).

‖ Two-sided raw P-values.

Supplementary Table 9. Primers for respiratory pathogen testing

Pathogen	Target gene	Primer name	Primer/Probe Sequence
RSV A	Nucleoprotein	RSVA Fwd.	AGA TCA ACT TCT GTC ATC CAG CAA
		RSVA Rev.	TTC TGC ACA TCA TAA TTA GGA GTA TCA AT
		RSVA Probe	CAC CAT CCA ACG GAG CAC AGG AGA T
RSV B	Nucleoprotein	RSVB Fwd.	AAG ATG CAA ATC ATA AAT TCA CAG GA
		RSVB Rev.	TGA TAT CCA GCA TCT TTA AGT ATC TTT ATA GTG
		RSVB Probe	AGG TAT GTT ATA TGC TAT GTC CAG GTT AGG AAG GGA A
RSV A*	Nucleoprotein	LTRSVA Fwd.	GATACACTCAACAAAGATCAACTTCTGTCA
		LTRSVA Rev.	AGGAGTGTCAATGCTGTCTCCTGTG
		LT RSVA Probe	TCCAGCAAATACACCATCCAACGGAG
RSV B*	Nucleoprotein	LTRSVB Fwd.	CTGTGTATAGCTGCCCTTGTAATAACC
		LTRSVB Rev.	GACATTGTTTGCCCTCCTAATTACTGC
		LTRSVB Probe	AGCAGCAGGAGATAGATCAGGTCTTACA
Influenza A	Matrix	FluA Fwd.	GAC CRA TCC TGT CAC CTC TGA
		FluA Rev.	AGG GCA TTY TGG ACA AAK CGT CTA
		FluA Probe	TGC AGT CCT CGC TCA CGT GGC ACG
Influenza B	Nucleoprotein	FluB Fwd.	AAG ACC TRA GAG TTT TGT CTG CAY T
		FluB Rev.	ATC AGA GCT GCY CCC ATT
		FluB Probe	TGC AAG GGT TTC CAY GTT CCA GCA
Parainfluenza-1	Polymerase	PIV-1 Fwd.	ACA GAT GAA ATT TTC AAG TGC TAC TTT AGT
		PIV-1 Rev.	GCC TCT TTT AAT GCC ATA TTA TCA TTA GA
		PIV-1 Probe	ATG GTA ATA AAT CGA CTC GCT
Parainfluenza-2	Polymerase	PIV-2 Fwd.	TGC ATG TTT TAT AAC TAC TGA TCT TGC TAA
		PIV-2 Rev.	GTT CGA GCA AAA TGG ATT ATG GT
		PIV-2 Probe	ACT GTC TTC AAT GGA GAT AT
Parainfluenza-3	Nucleoprotein	PIV-3 Fwd.	TGT TGA GCC TAT TTG ATA CAT TTA ATG C
		PIV-3 Rev.	ATG ATA GCT CCA CCA GCT GAT TTT
		PIV-3 Probe	CGT AGG CAA GAA AAC ATA A
Rhinovirus	5'UTR	HRV Fwd	CY+ AGC C+T GCG TGG C (+ is LNA base)
		HRV Rev.	GAA ACA CGG ACA CCC AAA GTA
		HRV Probe	TCC TCC GGC CCC TGA ATG YGG C
Human Metapneumovirus	Nucleoprotein	HMPV Fwd.	CAT ATA AGC ATG CTA TAT TAA AAG AGT CTC
		HMPV Rev.	CCT ATT TCT GCA TAT TTG TAA TCA G
		HMPV Probe	TGY AAT GAT GAG GGT GTC ACT GCG GTT G

Enterovirus	5'UTR	Ent Fwd.	GAT TGT CAC CAT AAG CAG C
		Ent Rev	CCC CTG AAT GCG GCT AAT C
		Ent Probe	CGG AAC CGA CTA CTT TGG GTG TCC GT
Human Corona virus 229E	Nucleoprotein	229E Fwd.	TCT GCC AAG AGT CTT GCT CG
		229E Rev.	AGC ATA GCA GCT GTT GAC GG
		229E Probe	TGG CCA CAA CAC CTG CAC TTC C
Human Corona virus OC43	Nucleoprotein	OC43 Fwd.	CAT CAG GAG GGA ATG TTG TAC C
		OC43 Rev.	TAC TGG TCT TTA GCA TGC GGT C
		OC43 Probe	CAG CAG TTG ACG CTG GTT GCC ATC
Human Corona virus HKU1	Replicase 1B	HKU1 Fwd.	CCT TGC GAA TGA ATG TGC T
		HKU1 Rev.	TTG CAT CAC CAC TGC TAG TAC CAC
		HKU1 Probe	TGT GTG GCG GTT GCT ATT ATG TTA AGC CTG
Human Corona virus NL63	Nucleoprotein	NL63 Fwd.	GAC CAA AGC ACT GAA TAA CAT TTT CC
		NL63 Rev.	ACC TAA TAA GCC TCT TTC TCA ACC C
		NL63 Probe	AAC ACG CTT CCA ACG AGG TTT CTT CAA CTG AG
Adenovirus	Hexon	Adeno Fwd.	CAG GAC GCC TCG GRG TAY CTS AG
		Adeno Rev.	GGA GCC ACV GTG GGR TT
		Adeno Probe	CCG GGT CTG GTG CAG TTT GCC C
Bocavirus	Non-structural protein 1	Boca Fwd.	TGC AGA CAA CGC YTA GTT GTT T
		Boca Rev.	CTG TCC CGC CCA AGA TAC A
		Boca Probe	CCA GGA TTG GGT GGA ACC TGC AAA
Mycoplasma pneumoniae	CARDS toxin	Myco Fwd.	TTT GGT AGC TGG TTA CGG GAA T
		Myco Rev.	GGT CGG CAC GAA TTT CAT ATA AG
		Myco Probe	TGT ACC AGA GCA CCC CAG AAG GGC T
Bordetella pertussis (Screening assay)	IS481	ISBordo Fwd.	GCG TGC AGA TTC GTC GTA C
		ISBordo Rev.	TGA TGG TGC CTA TTT TAC GG
		ISBordo Probe	ACC CTC GAT TCT TCC GT
Bordetella pertussis (Confirmatory assay)	Toxin	Bordo Fwd.	GCG TGC AGA TTC GTC GTA C
		Bordo Rev.	AGG GCA TTY TGG ACA AAK CGT CTA
		Bordo Probe	TGC AGT CCT CGC TCA CTG GGC ACG

Supplementary Table 10. Chromatography condition for metabolome profiling

Chromatography condition	Positive ionization chromatographically optimized for hydrophilic compounds	Positive ionization chromatographically optimized for hydrophobic compounds	Negative ionization optimized conditions	Negative ionization with HILIC chromatography
Column	Waters UPLC BEH C18-2.1x100 mm, 1.7 µm	Waters UPLC BEH C18-2.1x100 mm, 1.7 µm	Waters UPLC BEH C18-2.1x100 mm, 1.7 µm	Waters UPLC BEH Amide 2.1x150 mm, 1.7 µm
Mobile phase A	0.05% PFPA in water, pH ~2.5 and 0.1% formic acid	0.05% PFPA in water, pH ~2.5 and 0.1% formic acid	6.5 mM ammonium bicarbonate in water, pH 8	10 mM ammonium formate in 15% water/ 5% methanol/ 80% acetonitrile (effective pH 10.16 with NH_4OH)
Mobile phase B	0.1% formic acid and 0.05% PFPA in methanol, pH ~2.5	0.1% formic acid and 0.05% PFPA in 50% methanol/ 50% acetonitrile, pH ~2.5	6.5 mM ammonium bicarbonate in 95% methanol/ 5% water	10 mM ammonium formate in 50% water/ 50% acetonitrile (effective pH 10.80 with NH_4OH)
Flow rate	0.35 mL/min	0.60 mL/min	0.35 mL/min	0.50 mL/min
Gradient elution	Linear gradient from 5% B to 80% B over 3.35 minutes	Linear gradient from 40 % B to 99.5% B over 1.0 minute, hold 99.5% B for 2.4 minutes.	Linear gradient from 0.5 to 70% B over 4.0 minutes, then rapid gradient to 99% B in 0.5 minutes	Linear gradient from 5% B to 50% B over 3.5 minutes, then linear gradient from 50% B to 95% B in 2 minutes
Spray voltage (V)	4000	4200	3300	3000
Mass range (m/z)	70-1000	110-1000	80-1000	80-1000
Sheath gas (au)	70	35	70	60
Auxiliary gas (au)	35	35	15	20
Source temp (°C)	300	400	300	300
Ion transfer tube temp (°C)	250	320	250	250
Norm collision energy (au)	52, 65, 78	52, 65, 78	52, 65, 78	48, 60, 72
MS AGC target (au)	1.00E+06	1.00E+06	1.00E+06	1.00E+06
MS Max Fill Time (ms)	60	60	60	60
MS^n Ion Target (au)	2.00E+05	2.00E+05	2.00E+05	2.00E+05
MS^n Max Fill Time (ms)	120	120	120	120

MS^n Isolation Window (m/z)	3	3	3	3
MS^n Dynamic Exclusion Time (s)	3	3	3	3
S-Lens RF Level	40	50	40	25

Abbreviations: MS, mass spectroscopy; HILIC, hydrophilic interaction liquid chromatography; PFPA, perfluoropentanoic acid; UPLC, ultra-high-performance liquid chromatography; *m/z*, mass-to-charge ratio

Supplementary Table 11. Principal investigators at the 17 participating sites in MARC-35

Amy D. Thompson, MD	Alfred I. duPont Hospital for Children, Wilmington, DE
Federico R. Laham, MD, MS	Arnold Palmer Hospital for Children, Orlando, FL
Jonathan M. Mansbach, MD, MPH	Boston Children's Hospital, Boston, MA
Vincent J. Wang, MD, MHA and Susan Wu, MD	Children's Hospital of Los Angeles, Los Angeles, CA
Michelle B. Dunn, MD and Jonathan M. Spergel, MD, PhD	Children's Hospital of Philadelphia, Philadelphia, PA
Juan C. Celedón, MD, DrPH	Children's Hospital of Pittsburgh, Pittsburgh, PA
Michael R. Gomez, MD, MS-HCA and Nancy Inhofe, MD	The Children's Hospital at St. Francis, Tulsa, OK
Brian M. Pate, MD and Henry T. Puls, MD	The Children's Mercy Hospital & Clinics, Kansas City, MO
Stephen J. Teach, MD, MPH	Children's National Medical Center, Washington, D.C.
Richard T. Strait, MD and Stephen C. Porter, MD, MSc, MPH	Cincinnati Children's Hospital and Medical Center, Cincinnati, OH
Ilana Y. Waynik, MD	Connecticut Children's Medical Center, Hartford, CT
Sujit Iyer, MD	Dell Children's Medical Center of Central Texas, Austin, TX
Michelle D. Stevenson, MD, MS	Norton Children's Hospital, Louisville, KY
Wayne G. Shreffler, MD, PhD and Ari R. Cohen, MD	Massachusetts General Hospital, Boston, MA
Anne K. Beasley, MD and Cindy S. Bauer, MD	Phoenix Children's Hospital, Phoenix, AZ
Thida Ong, MD and Markus Boos, MD, PhD	Seattle Children's Hospital, Seattle, WA
Charles G. Macias, MD, MPH	Texas Children's Hospital, Houston, TX

Supplementary Fig. 1. Study flow diagram

The differences in the analytic and non-analytic cohorts are summarized in **Supplementary Table 1**.
* The microbiome and transcriptome data are obtained in 221 infants who were randomly-selected from the longitudinal cohort.

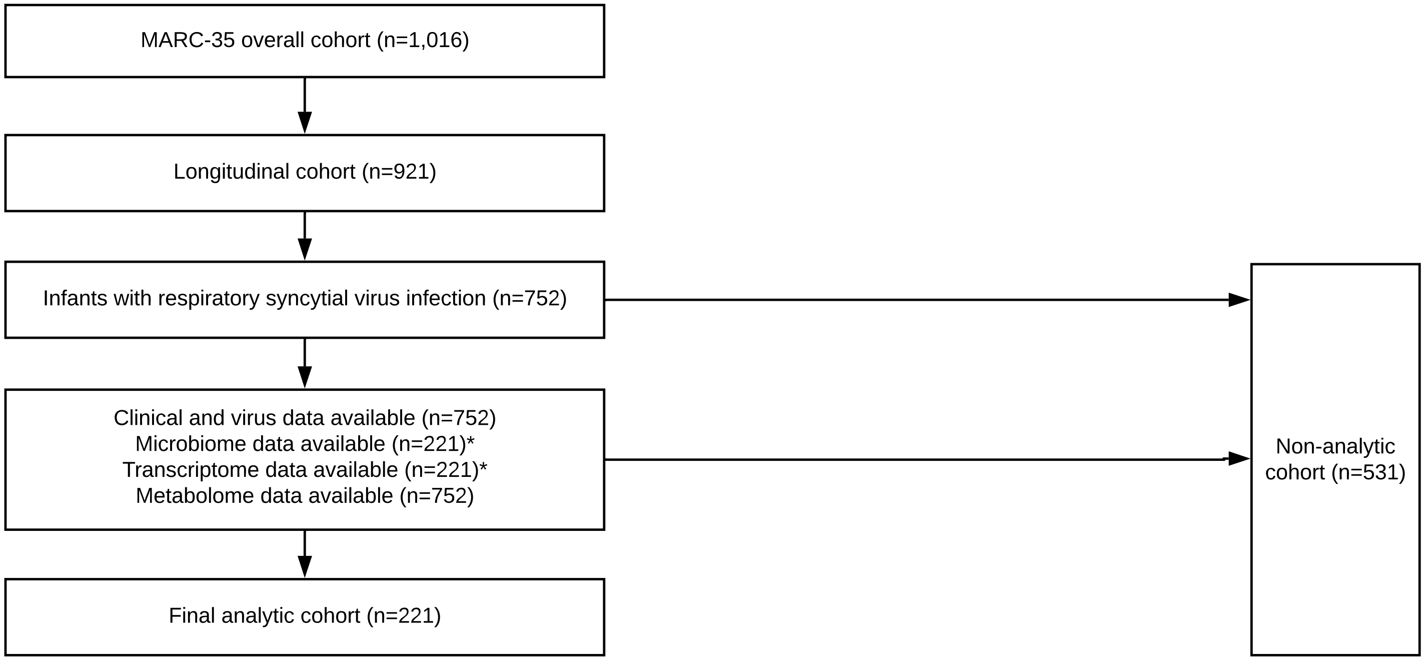

Supplementary Fig. 2. Average silhouette score and network modularity, according to number of endotypes

a. Average silhouette score, according to number of endotypes

Across the different numbers of endotypes (*k* of 2-6), the average silhouette score was highest with *k*=4.

b. Network modularity, according to number of endotypes

Across the different numbers of endotypes (*k* of 2-6), the network modularity was highest with *k*=4.

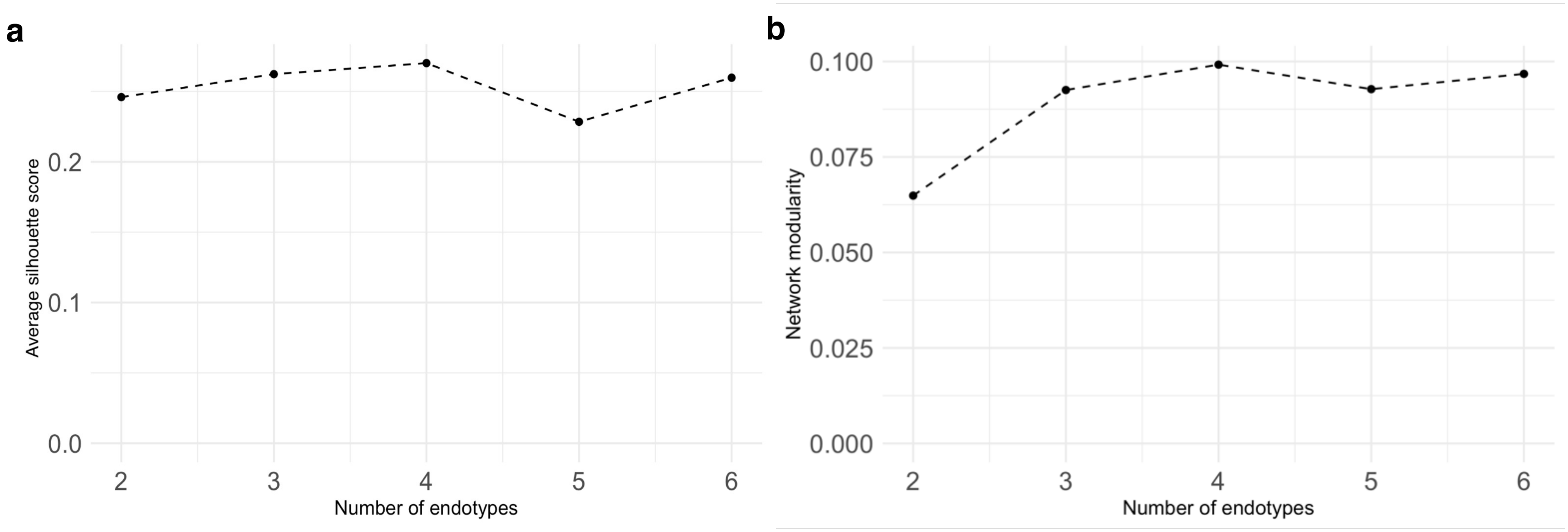

Supplementary Fig. 3. Similarity network visualization of respiratory syncytial virus bronchiolitis endotypes

The goal of this figure is a network visualization. We have applied integrative network and clustering approaches to clinical, virus, nasopharyngeal airway microbiome, transcriptome, and metabolome data. This network-based clustering method identifies distinct endotypes based on the degree of connectivity. In other words, infants with similar clinical and biological characteristics are more-closely connected with each other based on the degree of clinical and biological similarity (Fig. 1), resulting in the formation of a connected component (i.e., an endotype) in the network.

Nodes (circles) with a same colour represent infants with a corresponding endotype (A, B, C, or D). Network graphs in the integrated omics similarity network are visualized using with Fruchterman-Reingold layout. Layout of the network from the four datasets are fixed with the position of integrated omics similarity network. We selected 1,500 edges with the highest similarity for the network visualization and the width of edge reflects the strength of similarity.

Abbreviations: RSV, respiratory syncytial virus; IFN, interferon; IL, interleukin

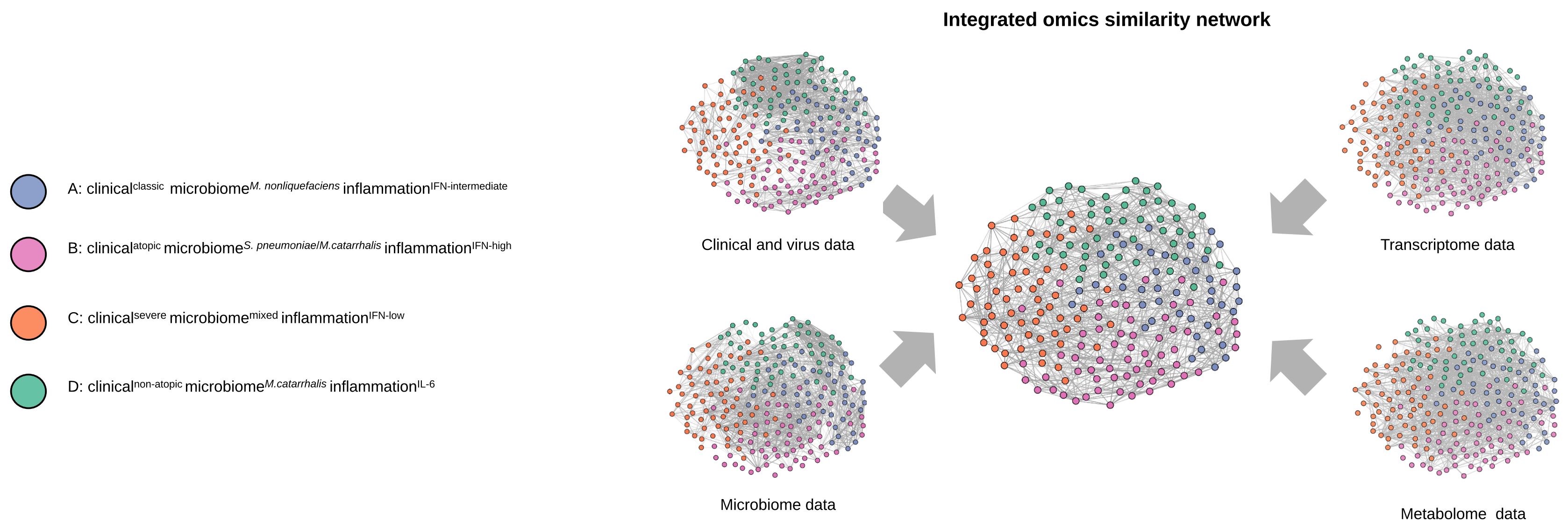

Integrated omics similarity network
Clinical and virus data
Transcriptome data
Microbiome data
Metabolome data
A: clinical^{classic} microbiome$^{M. nonliquefaciens}$ inflammation$^{IFN\text{-}intermediate}$
B: clinical^{atopic} microbiome$^{S. pneumoniae/M.catarrhalis}$ inflammation$^{IFN\text{-}high}$
C: clinical^{severe} microbiome^{mixed} inflammation$^{IFN\text{-}low}$
D: clinical$^{non\text{-}atopic}$ microbiome$^{M.catarrhalis}$ inflammation$^{IL\text{-}6}$

Supplementary Fig. 4. Differential gene expression analysis and functional pathway analysis in the endotype A vs. C comparison

a. Heatmap and volcano plot of differentially-expressed genes

For the heatmap (left), we selected 300 genes with the most significant P-value (two-sided raw P-value) and the colour bar indicates the scaled value of variance stabilizing transformation. For the volcano plot (right), the threshold of $\log_2$ fold change is |0.58| (i.e., $\geq |1.5|$-fold change) and that of FDR is 0.1. There were 21 differentially-expressed genes that met these criteria. Of these, 12 Ensemble gene ids are annotated in the volcano plot due to space availability.

b. Functional pathway analysis

For the functional class scoring analysis (left), we selected 25 pathways with the highest absolute value of normalized enriched score to visualize the plot.

c. Wilcoxon pathway enrichment analysis integrating transcriptome and metabolome data

For the Wilcoxon pathway enrichment analysis, we selected 20 pathways with the most significant joint FDR, and showed the numbers and proportions of hit genes (left) and metabolites (right) for the corresponding pathways.

Abbreviations: GSEA, gene set enrichment analysis; FDR, false discovery rate

Comparison of endotype A (reference) with endotype C

a Heatmap and volcano plot of differentially-expressed genes

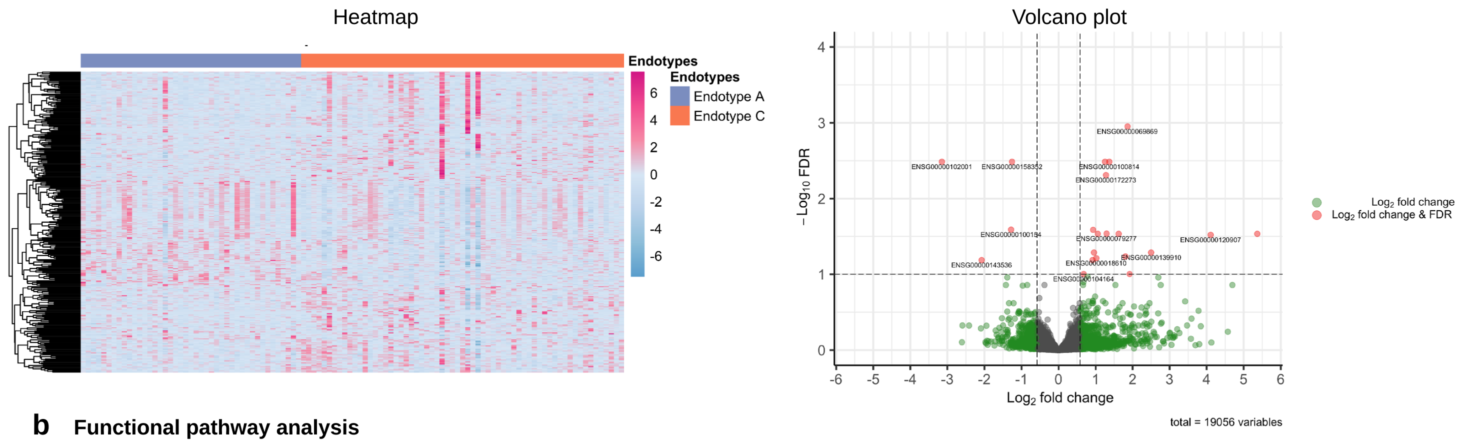

b Functional pathway analysis

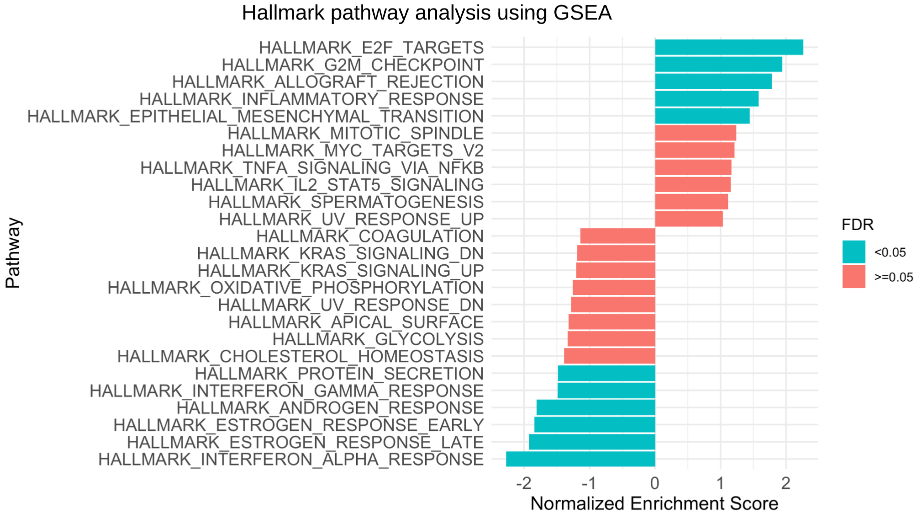

c Wilcoxon pathway enrichment analysis integrating transcriptome and metabolome data

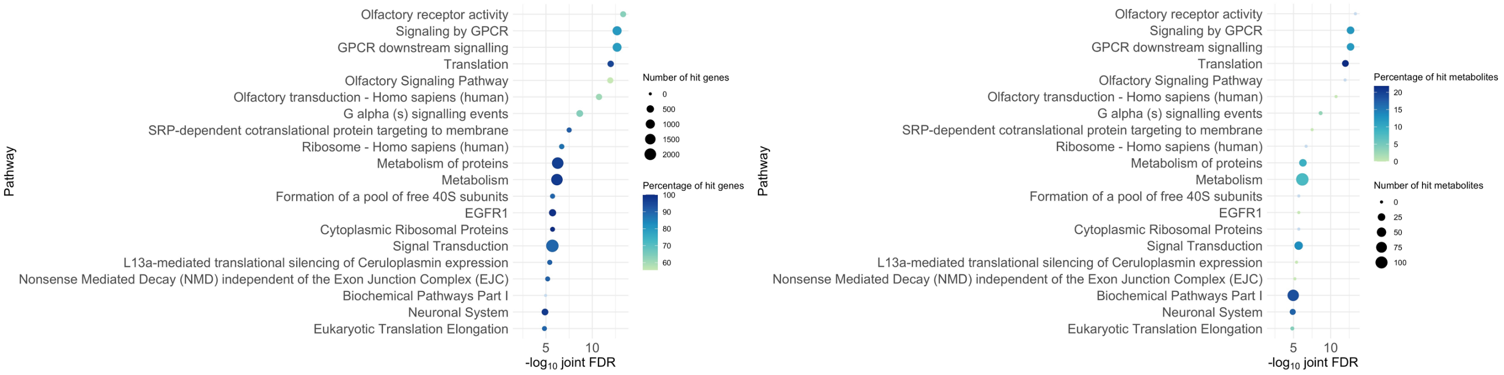

Supplementary Fig. 5. Differential gene expression analysis and functional pathway analysis in the endotype A vs. D comparison

a. Heatmap and volcano plot of differentially-expressed genes

For the heatmap (left), we selected 300 genes with the most significant P-value (two-sided raw P-value) and the colour bar indicates the scaled value of variance stabilizing transformation. For the volcano plot (right), the threshold of $\log_2$ fold change is |0.58| (i.e., ≥ |1.5|-fold change) and that of FDR is 0.1. There were 65 differentially-expressed genes that met these criteria. Of these, 18 Ensemble gene ids are annotated in the volcano plot due to space availability.

b. Functional pathway analysis

For the functional class scoring analysis (left), we selected 25 pathways with the highest absolute value of normalized enriched score to visualize the plot.

c. Wilcoxon pathway enrichment analysis integrating transcriptome and metabolome data

For the Wilcoxon pathway enrichment analysis, we selected 20 pathways with the most significant joint FDR, and showed the numbers and proportions of hit genes (left) and metabolites (right) for the corresponding pathways.

Abbreviations: GSEA, gene set enrichment analysis; FDR, false discovery rate

Comparison of endotype A (reference) with endotype D

a **Heatmap and volcano plot of differentially-expressed genes**

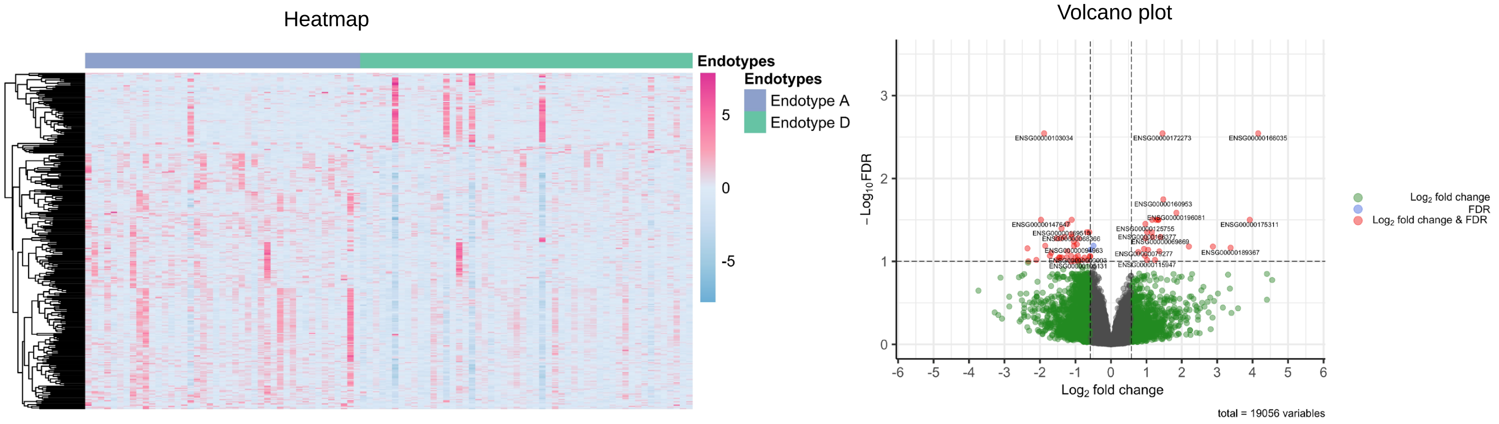

b **Functional pathway analysis**

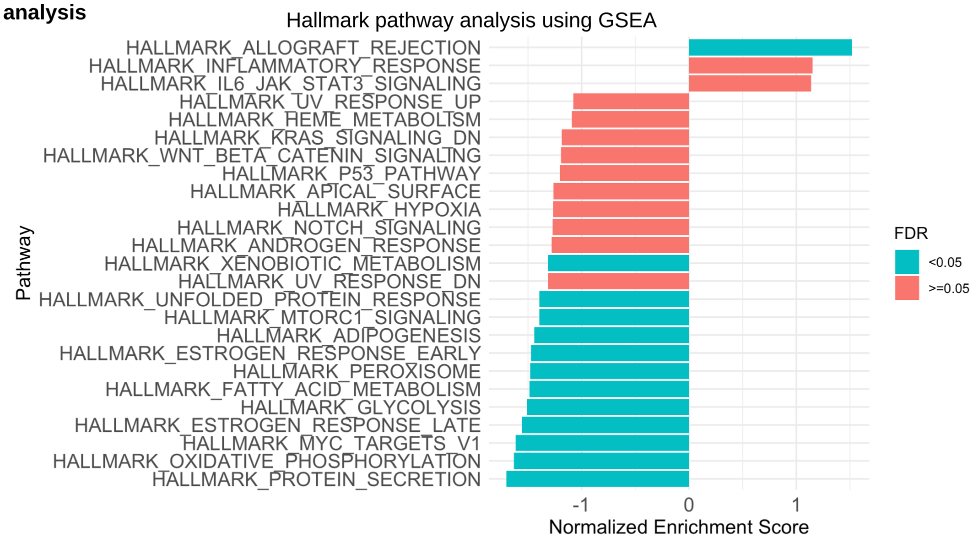

c **Wilcoxon pathway enrichment analysis integrating transcriptome and metabolome data**

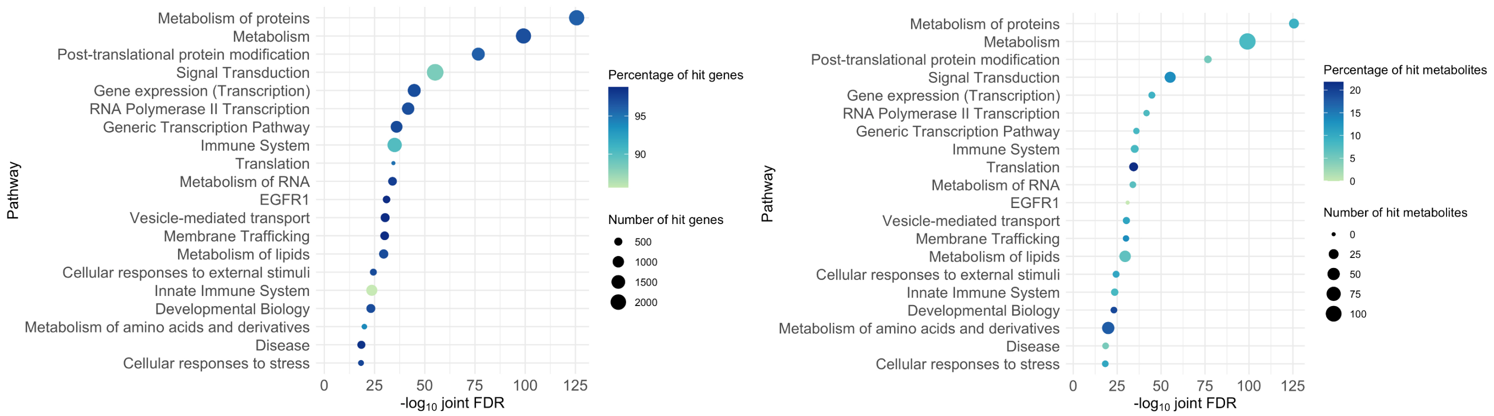

Supplementary Fig. 6. Ranking of normalized mutual information score

The ranking of normalized mutual information score of each dataset is shown in the four plots: **a.** clinical and virus data (all variables used for calculating Gower distance); **b.** nasopharyngeal microbiome data (top 20 species); **c.** transcriptome data (top 30 transcripts); and **d.** metabolome data (top 30 metabolites).

Abbreviations: RSV, respiratory syncytial virus; IgE, immunoglobulin E; BHBA, beta-Hydroxybutyric acid; GPE, glycerophosphoethanolamine; GPG, glycerophosphoglycerol; GPI, Glycosylphosphatidylinositol; GPS, glycerophosphatidylserine; GPC, glycerophosphorylcholine

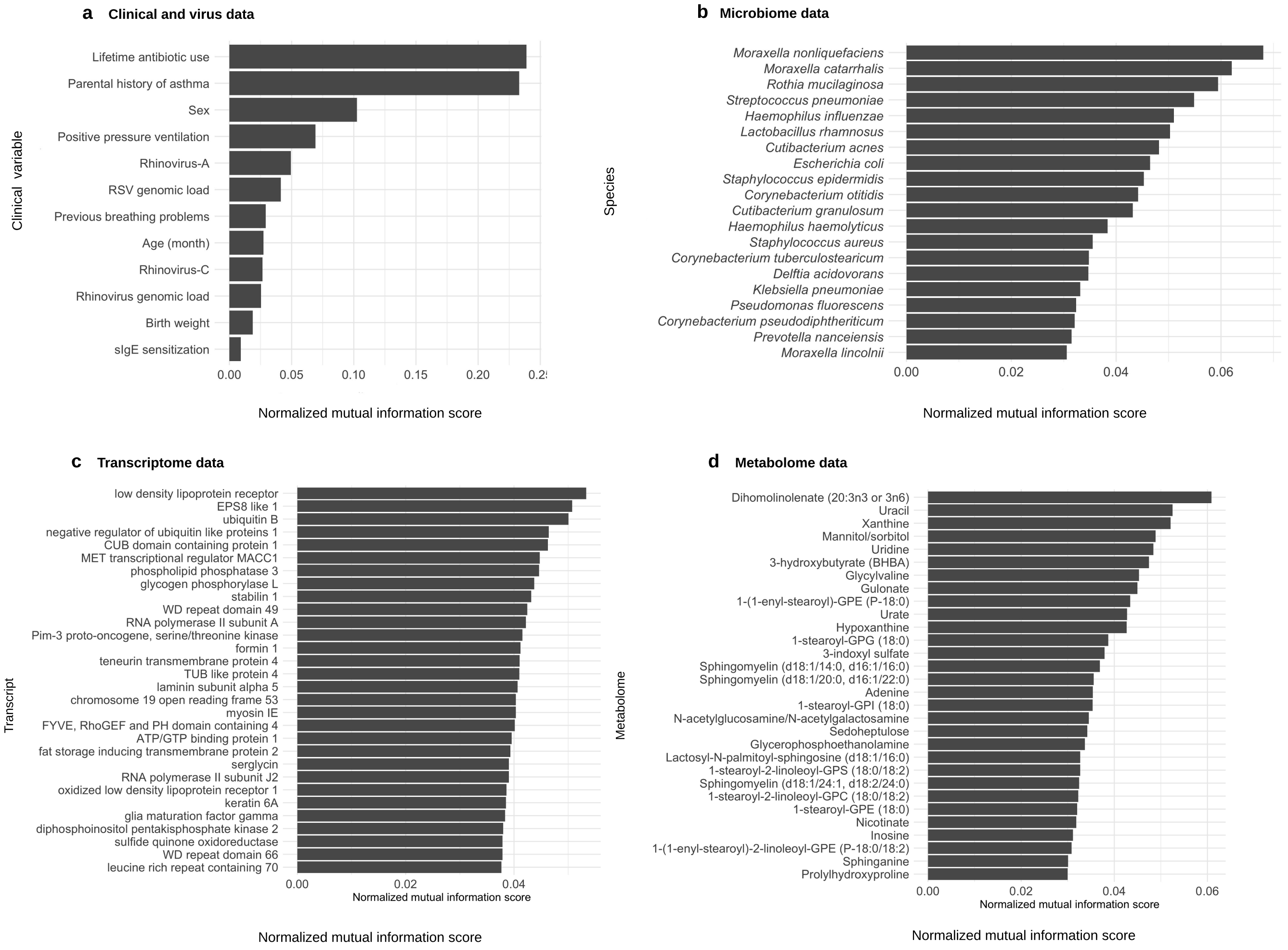

Supplementary Fig. 7. Alluvial plot that examines consistencies between original analysis and analysis using all variables

Each color band represents a group of infants with an original endotype (A-D). There are consistencies between the four original endotypes (A-D) and four endotypes using all variables. Rand index representing the statistics of concordance between two clustering results is 0.87 (95%CI, 0.83-0.90).

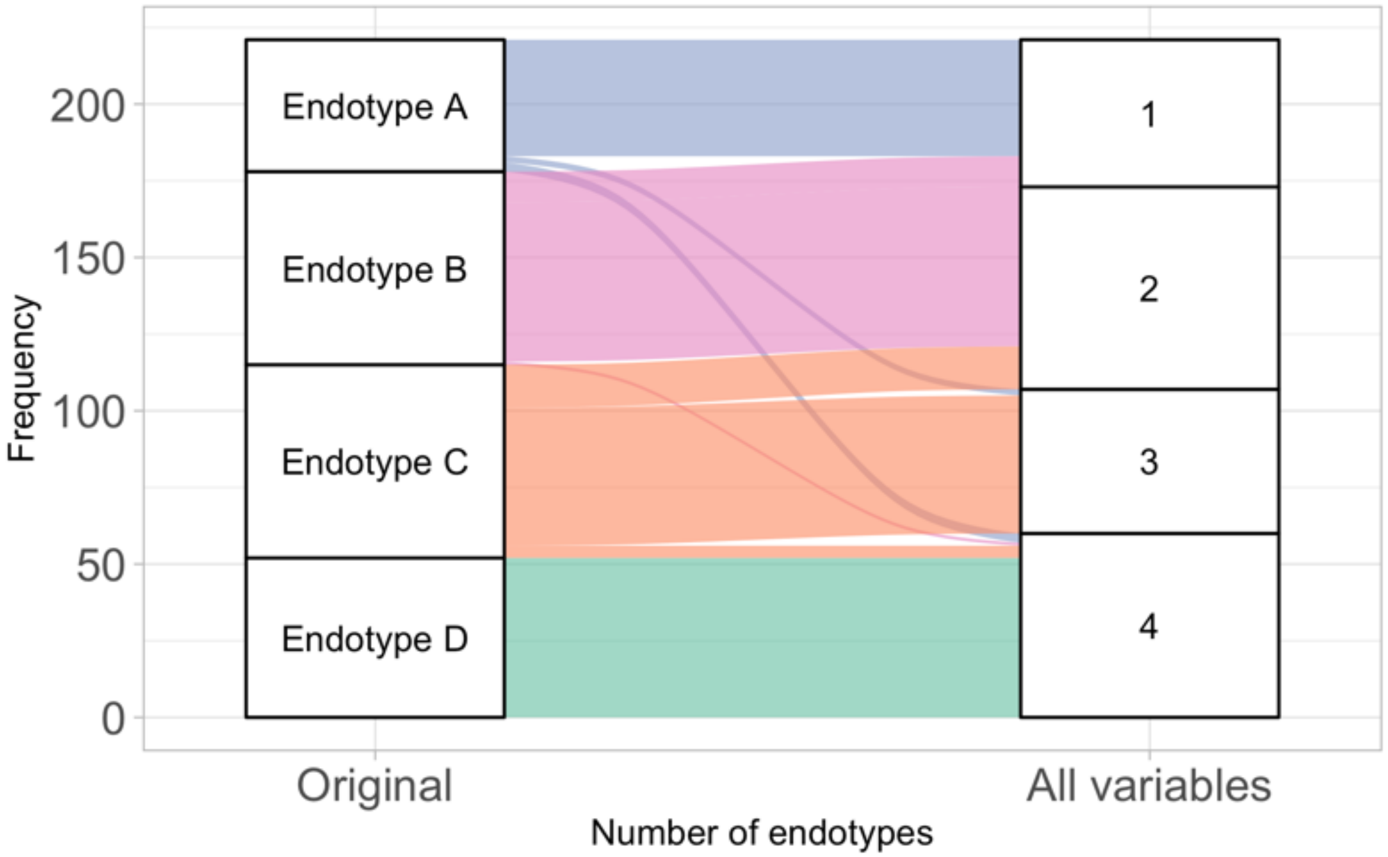

Supplementary Fig. 8. Alluvial plot that examines consistencies across different number of endotypes

Each color band represents a group of infants with an original endotype (A-D). There are consistencies between the four original endotypes (A-D) and three and five endotypes, particularly those with an endotype A (reference) or B. The numbers of infants assigned to each endotype are presented in Supplementary Table 7.

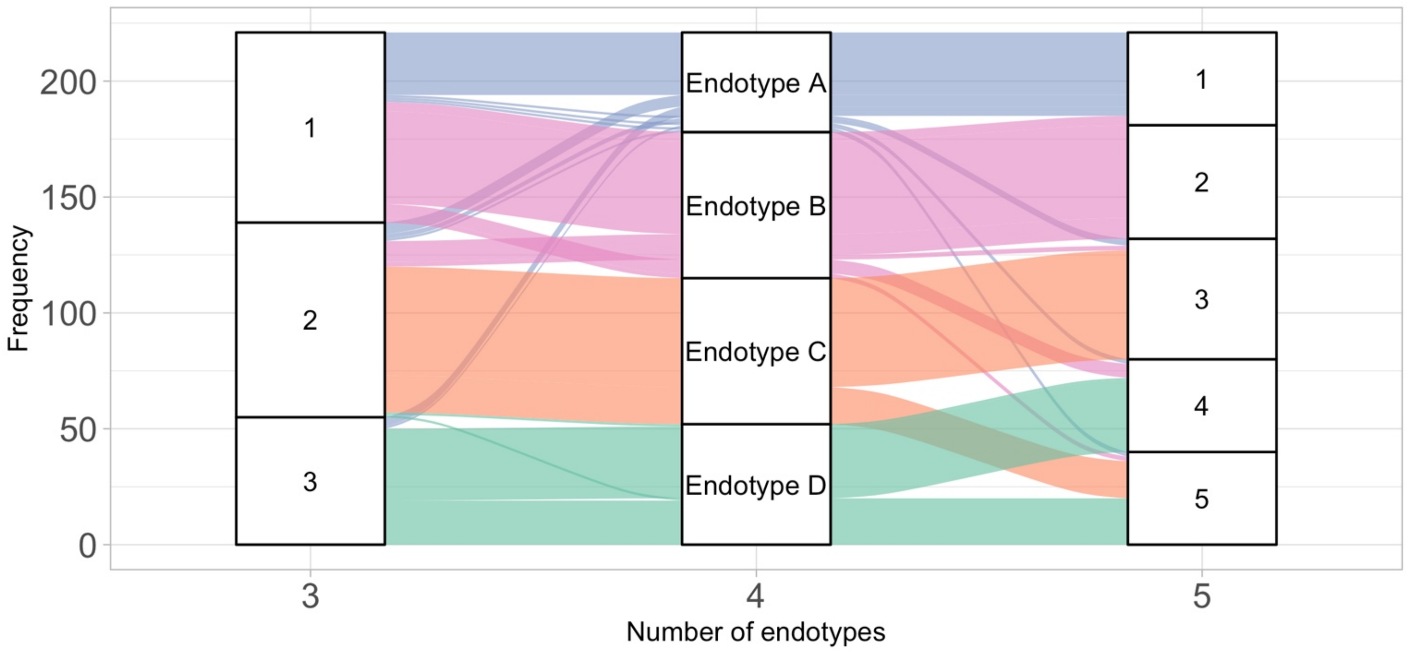

Supplementary Fig. 9. Differential gene expression analysis and functional pathway analysis in the endotype 1 vs. 2 comparison in the sensitivity analysis using five endotypes (corresponding to endotype A vs B comparison in the primary analysis)

a. Heatmap and volcano plot of differentially-expressed genes

For the heatmap (left), we selected 300 genes with the most significant P-value (two-sided raw P-value) and the colour bar indicates the scaled value of variance stabilizing transformation. For the volcano plot (right), the threshold of $\log_2$ fold change is |0.58| (i.e., ≥ |1.5|-fold change) and that of FDR is 0.1. There were 6 differentially-expressed genes that met these criteria.

b. Functional pathway analysis

For the functional class scoring analysis, we selected 25 pathways with the highest absolute value of normalized enriched score to visualize the plot.

c. Wilcoxon pathway enrichment analysis integrating transcriptome and metabolome data

For the Wilcoxon pathway enrichment analysis, we selected 20 pathways with the most significant joint FDR, and showed the numbers and proportions of hit genes (left) and metabolites (right) for the corresponding pathways.

Abbreviations: GSEA, gene set enrichment analysis; FDR, false discovery rate

Comparison of endotype 1 (reference [corresponding to endotype A) with endotype 2 (corresponding to endotype B)

a Heatmap and volcano plot of differentially-expressed genes

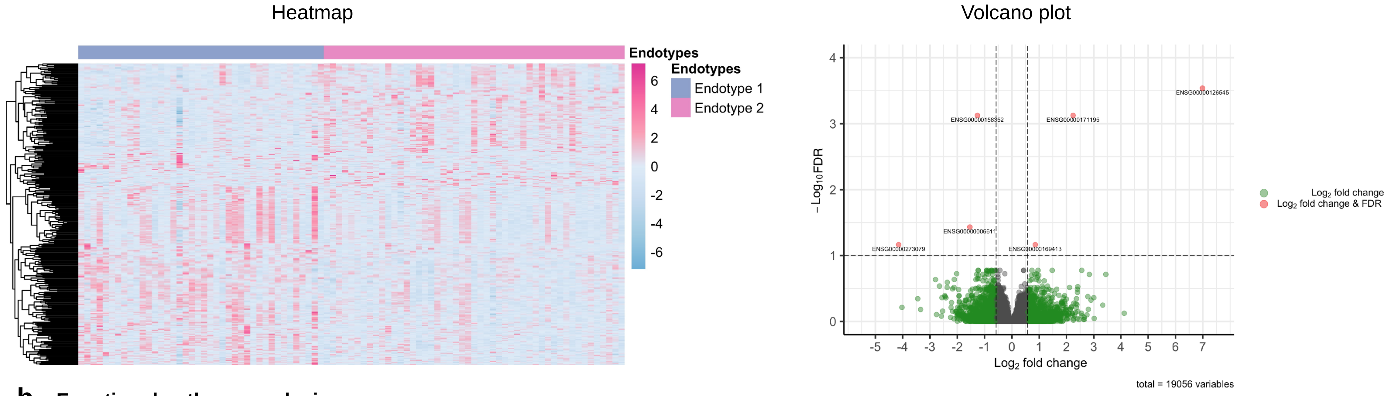

b Functional pathway analysis

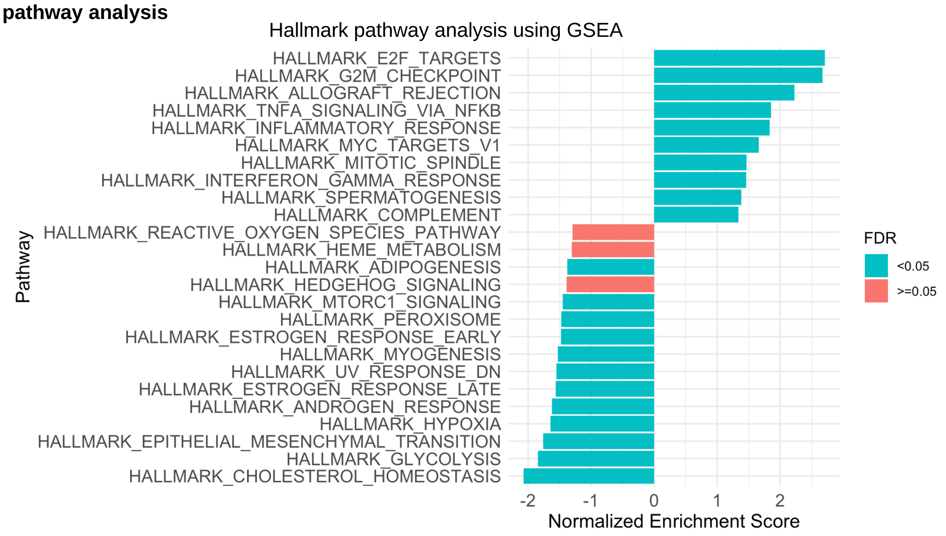

c Wilcoxon pathway enrichment analysis integrating transcriptome and metabolome data

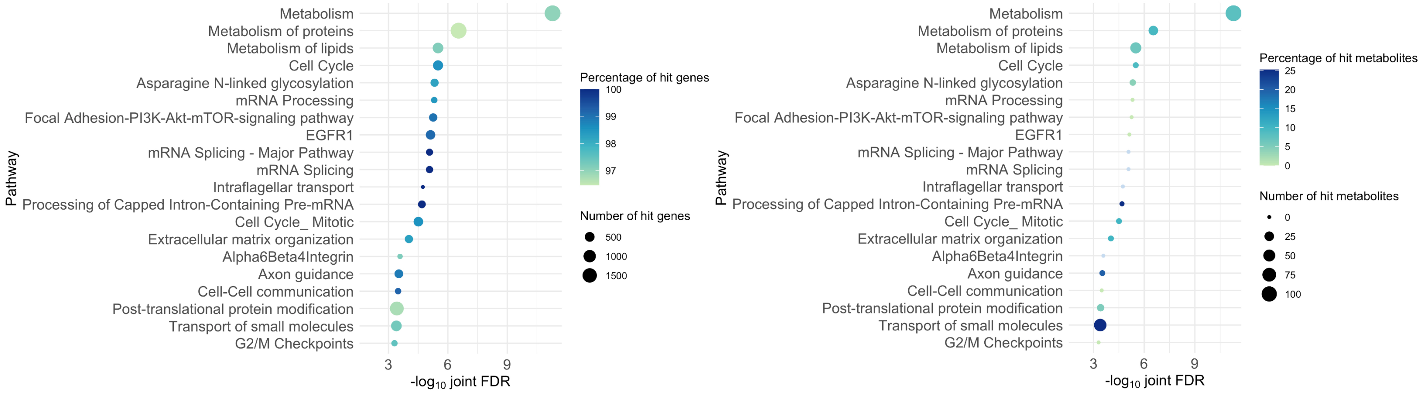

Supplement: Supplementary file 1 — Supplementary Information [file 41467_2021_23859_MOESM1_ESM.pdf]
