# Supplementary material for: Integrated omics endotyping of infants with respiratory syncytial virus bronchiolitis and risk of childhood asthma
Source: Nat Commun. 2021 Jun 14;12:3601. doi: 10.1038/s41467-021-23859-6 (PMC8203688; doi:10.1038/s41467-021-23859-6)
Supplement: Supplementary file 3 — Reporting Summary [file 41467_2021_23859_MOESM3_ESM.pdf]

nature research

Corresponding author(s): Yoshihiko Raita

Last updated by author(s): 4/30/2021

Reporting Summary

Nature Research wishes to improve the reproducibility of the work that we publish. This form provides structure for consistency and transparency in reporting. For further information on Nature Research policies, see our Editorial Policies and the Editorial Policy Checklist.

Statistics

For all statistical analyses, confirm that the following items are present in the figure legend, table legend, main text, or Methods section.

n/a	Confirmed	
☐	☒	The exact sample size (*n*) for each experimental group/condition, given as a discrete number and unit of measurement
☐	☒	A statement on whether measurements were taken from distinct samples or whether the same sample was measured repeatedly
☐	☒	The statistical test(s) used AND whether they are one- or two-sided<br>*Only common tests should be described solely by name; describe more complex techniques in the Methods section.*
☐	☒	A description of all covariates tested
☐	☒	A description of any assumptions or corrections, such as tests of normality and adjustment for multiple comparisons
☐	☒	A full description of the statistical parameters including central tendency (e.g. means) or other basic estimates (e.g. regression coefficient) AND variation (e.g. standard deviation) or associated estimates of uncertainty (e.g. confidence intervals)
☐	☒	For null hypothesis testing, the test statistic (e.g. *F*, *t*, *r*) with confidence intervals, effect sizes, degrees of freedom and *P* value noted<br>*Give P values as exact values whenever suitable.*
☒	☐	For Bayesian analysis, information on the choice of priors and Markov chain Monte Carlo settings
☒	☐	For hierarchical and complex designs, identification of the appropriate level for tests and full reporting of outcomes
☒	☐	Estimates of effect sizes (e.g. Cohen's *d*, Pearson's *r*), indicating how they were calculated

Our web collection on statistics for biologists contains articles on many of the points above.

Software and code

Policy information about availability of computer code

Data collection	REDCap: v10.0.30

Data analysis	R version 3.6.1<br>R missForest (v1.4) package<br>R DESeq2 Release (v3.12)<br>R sva package Release (v3.12)<br>R StatMatch (v1.4.0) package<br>R vegan (v2.5-7) package<br>R amap (v0.8-18) package<br>R SNFtool (v2.3.0) package<br>R qgraph (v1.6.9) package<br>R circlize (v0.4.12) package<br>R VennDiagram (v1.6.20) package<br>R ComplexUpset (v1.2.1) package<br>R fgsea Release (v3.12)<br><br>PathoScope (v2.0) used in this study can be found in the following site: https://github.com/PathoScope/PathoScope<br>Salmon (v1.4.0) used in this study can be found in the following site: https://combine-lab.github.io/salmon/<br>QUICS is a software owned by Metabolon<br>Integrated Molecular Pathway Level Analysis (IMPaLA): Release 34 (15.01.2019) -- publicly available in http://impala.molgen.mpg.de/

For manuscripts utilizing custom algorithms or software that are central to the research but not yet described in published literature, software must be made available to editors and reviewers. We strongly encourage code deposition in a community repository (e.g. GitHub). See the Nature Research guidelines for submitting code & software for further information.

Data

Policy information about availability of data

All manuscripts must include a data availability statement. This statement should provide the following information, where applicable:

- Accession codes, unique identifiers, or web links for publicly available datasets
- A list of figures that have associated raw data
- A description of any restrictions on data availability

Data availability: All relevant data that support the findings of this study will be available on the NIH/NIAID ImmPort and/or dbGaP through controlled access or from the authors. To be compliant with the informed consent forms of MARC-35 study and the genomic data sharing plan, the data are available only for research that studies the possible genetic causes of severe bronchiolitis, recurrent wheezing, asthma and related concepts.

Field-specific reporting

Please select the one below that is the best fit for your research. If you are not sure, read the appropriate sections before making your selection.

☒ Life sciences ☐ Behavioural & social sciences ☐ Ecological, evolutionary & environmental sciences

For a reference copy of the document with all sections, see nature.com/documents/nr-reporting-summary-flat.pdf

Life sciences study design

All studies must disclose on these points even when the disclosure is negative.

Sample size	This is a secondary analysis of data from an observational study and the sample size of the final analytic cohort is 221. In this secondary analysis, a sample size calculation is not applicable. We have described the sample flow chart in the Supplementary Fig. 1. Regardless, we conducted a post-hoc power calculation based on the results. The power to detect the observed effect size (9% vs. 38%) is 93.9% in the endotype A vs. endotype B comparison (n=106).
Data exclusions	The original longitudinal cohort consisted of 921 infants. Of these, the microbiome and transcriptome data were obtained in 221 infants who were randomly-selected from the longitudinal cohort, contributing to the analytic cohort. We did not exclude any data who met the inclusion criteria.
Replication	We did not perform an external validation. This is the first study that has identified biologically-meaningful endotypes in infants with severe RSV bronchiolitis by integrating four different types of data and demonstrated their longitudinal relations with the risk of chronic morbidities. This study should facilitate further validation research.
Randomization	Not applicable. This is not a randomized trial. Besides, we do not have a "control" group. In this study, we have analyzed 221 infants with severe bronchiolitis.
Blinding	Not applicable. This is an observational study.

Reporting for specific materials, systems and methods

We require information from authors about some types of materials, experimental systems and methods used in many studies. Here, indicate whether each material, system or method listed is relevant to your study. If you are not sure if a list item applies to your research, read the appropriate section before selecting a response.

Materials & experimental systems

n/a	Involved in the study
x	Antibodies
x	Eukaryotic cell lines
x	Palaeontology and archaeology
x	Animals and other organisms
	x Human research participants
x	Clinical data
x	Dual use research of concern

Methods

n/a	Involved in the study
x	ChIP-seq
x	Flow cytometry
x	MRI-based neuroimaging

Human research participants

Policy information about studies involving human research participants

Population characteristics	This prospective cohort study completed enrollment of infants (age <1 year) hospitalized with bronchiolitis at 17 sites across 14 U.S. states. Of these 1,016 infants (median age, 3 months; female, 40%), 921 (91%) completed the run-in procedure (contact at both 1-week after hospital discharge or 3-weeks after hospitalization) and comprise the MARC-35 longitudinal cohort. Of the infants enrolled into this longitudinal cohort, the current study included 221 infants with RSV bronchiolitis who were randomly-selected for nasopharyngeal microbiome, transcriptome, and metabolome testing (Supplementary Fig. 1). The analytic cohort and non-analytic cohort did not differ in patient characteristics ($P \geq 0.05$; Supplementary Table 1), except for daycare use. Among the analytic cohort, the median age was 3 (IQR, 2-6) months, 42% were female, and 42% were non-Hispanic white. Overall, 72% were solo-RSV infection while 13% had coinfection with rhinovirus (Table 1).
Recruitment	Each morning from November 1 until April 30 of the study years, a member of the site research team screened all children admitted with bronchiolitis to the medical ward, any “intermediate care” type of unit, and the intensive care unit, in the past 24 hours. Using a variety of mechanisms (e.g. patient logs, communication with medical teams, computerized registry, on-site research assistants), site investigators recorded whether they were either approached, missed (no attempt to approach), or known to be ineligible from pre-screen of record in the screening form. We enrolled children, male and female, of all races.<br><br>Once the general inclusion criteria (age <1 year, admitted to hospital, physician diagnosis of bronchiolitis, parent/legal guardian’s ability to give informed consent) have been confirmed, study personnel approached the parent/legal guardian, related a brief overview of the study and asked if he or she is interested in hearing more about the study. If interested, study personnel administered the main section of the screening form to finally confirm eligibility. If the parent/legal guardian indicated a willingness to participate, the site investigator or study personnel obtained parent/legal guardian consent.<br><br>This study has some potential limitations with regards to the selection of participants. First, we focused on infants with RSV bronchiolitis while other non-RSV viruses are also causative pathogens. However, RSV not only contributes to 75% of severe bronchiolitis, but also has the largest population attributable fraction in asthma development. Second, the current study did not have healthy “controls”. Yet, the study objective was not to evaluate the difference of endotypes from healthy infants but to define endotypes of RSV bronchiolitis. Third, the study sample consisted of racially/ethnically- and geographically-diverse infants hospitalized for bronchiolitis. While our sample had a large severity contrast, our inferences may not be generalizable to infants in ambulatory settings with mild-to-moderate bronchiolitis and warrant external validation. Nonetheless, our data remain relevant for the 110,000 infants hospitalized yearly in the U.S., a vulnerable population with substantial morbidity burden.
Ethics oversight	The institutional review board at each of the participating hospitals approved the study. Written informed consent was obtained from the parent or guardian.<br>Principal investigators at the 17 participating sites in MARC-35 are the following:<br>Amy D. Thompson, MD Alfred I. duPont Hospital for Children, Wilmington, DE;<br>Federico R. Laham, MD, MS Arnold Palmer Hospital for Children, Orlando, FL;<br>Jonathan M. Mansbach, MD, MPH Boston Children's Hospital, Boston, MA;<br>Vincent J. Wang, MD, MHA and Susan Wu, MD Children's Hospital of Los Angeles, Los Angeles, CA;<br>Michelle B. Dunn, MD and Jonathan M. Spergel, MD, PhD Children's Hospital of Philadelphia, Philadelphia, PA;<br>Juan C. Celedón, MD, DrPH Children's Hospital of Pittsburgh, Pittsburgh, PA;<br>Michael R. Gomez, MD, MS-HCA and Nancy Inhofe, MD The Children's Hospital at St. Francis, Tulsa, OK;<br>Brian M. Pate, MD and Henry T. Puls, MD The Children's Mercy Hospital & Clinics, Kansas City, MO;<br>Stephen J. Teach, MD, MPH Children's National Medical Center, Washington, D.C.;<br>Richard T. Strait, MD and Stephen C. Porter, MD, MSc, MPH Cincinnati Children's Hospital and Medical Center, Cincinnati, OH;<br>Ilana Y. Waynik, MD Connecticut Children's Medical Center, Hartford, CT;<br>Sujit Iyer, MD Dell Children's Medical Center of Central Texas, Austin, TX;<br>Michelle D. Stevenson, MD, MS Norton Children's Hospital, Louisville, KY;<br>Wayne G. Shreffler, MD, PhD and Ari R. Cohen, MD Massachusetts General Hospital, Boston, MA;<br>Anne K. Beasley, MD and Cindy S. Bauer, MD Phoenix Children's Hospital, Phoenix, AZ;

Thida Ong, MD and Markus Boos, MD, PhD Seattle Children's Hospital, Seattle, WA;
Charles G. Macias, MD, MPH Texas Children's Hospital, Houston, TX;

Note that full information on the approval of the study protocol must also be provided in the manuscript.
